# Light-assisted delithiation of lithium iron phosphate nanocrystals towards photo-rechargeable lithium ion batteries

**DOI:** 10.1038/ncomms14643

**Published:** 2017-04-10

**Authors:** Andrea Paolella, Cyril Faure, Giovanni Bertoni, Sergio Marras, Abdelbast Guerfi, Ali Darwiche, Pierre Hovington, Basile Commarieu, Zhuoran Wang, Mirko Prato, Massimo Colombo, Simone Monaco, Wen Zhu, Zimin Feng, Ashok Vijh, Chandramohan George, George P. Demopoulos, Michel Armand, Karim Zaghib

**Affiliations:** 1 Institute de Recherche d-Hydro-Québec (IREQ), 1800 Boulevard Lionel Boulet, Varennes, Quebec, Canada J3X 1S1; 2 Department of Mining and Materials Engineering, McGill University, Wong Building, 3610 University Street, Montreal, Quebec, Canada H3A OC5; 3 IMEM-CNR, Parco Area delle Scienze 37/A, 43124 Parma, Italy; 4 Nanochemistry Department, Istituto Italiano di Tecnologia, via Morego 30, 16130 Genova, Italy; 5 Institute for Manufacturing, Department of Engineering, University of Cambridge, 17 Charles Babbage Road, Cambridge CB3 0FS, UK; 6 Cicenergigune Parque Tecnologico C/Albert Einstein 48 CP, 01510 Minano (Alava), Spain

## Abstract

Recently, intensive efforts are dedicated to convert and store the solar energy in a single device. Herein, dye-synthesized solar cell technology is combined with lithium-ion materials to investigate light-assisted battery charging. In particular we report the direct photo-oxidation of lithium iron phosphate nanocrystals in the presence of a dye as a hybrid photo-cathode in a two-electrode system, with lithium metal as anode and lithium hexafluorophosphate in carbonate-based electrolyte; a configuration corresponding to lithium ion battery charging. Dye-sensitization generates electron–hole pairs with the holes aiding the delithiation of lithium iron phosphate at the cathode and electrons utilized in the formation of a solid electrolyte interface at the anode via oxygen reduction. Lithium iron phosphate acts effectively as a reversible redox agent for the regeneration of the dye. Our findings provide possibilities in advancing the design principles for photo-rechargeable lithium ion batteries.

## Abstract (web summary)

Photo-rechargable energy storage cells can provide plug-free power for various applications. Here the authors integrate a photo-absorbing dye complex with LiFePO_4_ nanocrystals as a lithium-ion battery cathode in a two-electrode system demonstrating its photo-charging and galvanostatic discharging.

The design of a device that is simultaneously a solar energy convertor and a battery represents a paradigm-shifting energy storage concept that allows to charge a battery without any external power supply^1^,^2^. The first photo-rechargeable battery was proposed in 1976 by Hodes *et al*.^3^ using a three-electrode system composed of cadmium selenide/sulfur/silver sulfide (CdSe/S/Ag_2_S), followed in 1977 (ref. 4) by the ternary system *n*-cadmium selenide telluride/caesium sulfide/tin sulfide (CdSe_0.65_Te_0.35_/Cs_2_S_*x*_/SnS). In 1990, Kanbara *et al*.^5^ investigated a photo-reaction on a semiconductor silicon/silicon oxide (P-I aSi/SiO_*x*_) electrode using silver iodide tungstanate (Ag_6_I_4_WO_4_) and observed a photo-sensitizing effect on the surface of SiO_*x*_. More recently, a solar rechargeable battery consisting of a hybrid titania (TiO_2_)/poly(3,4-ethylenedioxythiophene, PEDOT) photo-anode and a perchlorate (ClO_4_^−^)-doped polypyrrole counter electrode was proposed by Liu *et al*. in 2012 (ref. 6). In 2014, Yu *et al*.^7^ reported charging of a lithium–oxygen (Li–O_2_) battery with the assistance of a redox-coupled dye photo-electrode. In the meantime, in 2015 Li *et al*.^8^ integrated a TiO_2_-based electrode in a three-electrode system comprising a lithium iron phosphate (LiFePO_4_; LFP)/lithium metal cell using triodide/iodide (I_3_^−^/I^−^) as a redox agent in a separate electrolyte compartment. All these devices are basically three-electrode systems that have two linked sections, namely: one dedicated to solar energy conversion and the other dedicated to energy storage as discussed recently by Li *et al*.^9^. Along the same lines, Xu *et al*.^10^ connected a perovskite methylammonium lead iodide (CH_3_NH_3_PbI_3_)-based solar cell in series with a Li-based cell (LFP cathode and a Li_4_Ti_5_O_12_ anode) and observed good cycling stability. Also in 2015, Thimmappa *et al*.^11^ proposed a chemically rechargeable photo-battery device utilizing potassium iron hexacyanoferrate prussian blue analogue (KFe[Fe(CN)_6_] and titanium nitride (TiN) in which: the photo-electrons generated on the TiN electrode assist in battery discharging while sodium disulphate Na_2_S_2_O_8_ participate in charging as is consumed and continuously regenerated. In another development, Li *et al*.^12^ proposed a very innovative device, integrating a CdSe@Pt photocatalyst into Li–S batteries via which direct solar energy storage takes place in the form of H_2_ production. In 2015, Yu *et al*.^13^ designed a photo-rechargeable Li-iodide flow battery, using a TiO_2_-dye photoelectrode via linkage of an I_3_^−^/I^−^ based catholyte for the conversion and storage of solar energy. Compared to the previous concepts, the devices described by Li and Wu are single systems. In Li's device, the electrons are consumed by the reduction of hydrogen (2H^+^+2e^−^→H_2_) while in Wu's device, a constant flow of a reversible I_3_^−^/I^−^ redox agent is required. For the two-electrode system, Liu *et al*.^14^ suggested in 2015 the use of a graphitic carbon nitride (C_3_N_4_) photocatalyst to reduce the charging voltage in a Li–O_2_ battery.

In this paper, we report a two-electrode system involving direct photo-oxidation of LFP nanocrystals by light irradiation in the presence of the N719 dye as hybrid photo-cathode, Li metal as anode, and LiPF_6_ organic carbonate solvent (EC/DEC/VC) as electrolyte that corresponds to Li-ion battery charging. We utilize LFP as the cathode material because of its stability and safety as well as its favourable redox potential. The latter, 3.4 V versus Li^+^/Li (refs 15, 16), is very close to that of the classic I_3_^−^/I^−^ redox couple (∼3.1 V versus Li^+^/Li) used in the dye-sensitized solar cell invented by O'Regan and Grätzel in 1991 (ref. 17). Dye-sensitization generates electron–hole pairs with the holes aiding the chemical conversion of LFP (triphylite) nanoplatelets to FePO_4_ (heterosite) at the cathode and the electrons utilized via oxygen reduction in the formation of solid electrolyte interface (SEI) at the anode made-up of lithium-carbonate-based species. The photo-assisted delithiation of LFP is reversible upon galvanostatic discharge. Our findings open possibilities in designing photo-rechargeable Li-ion batteries based on a two-electrode device configuration.

## Results

### Observation of LFP delithiation

**Figure 1:**
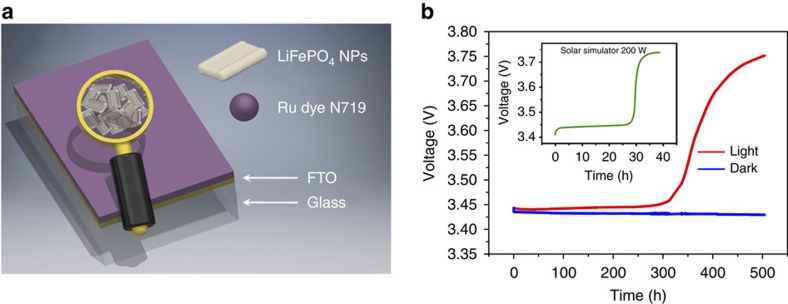
LiFePO_4_/Dye photocathode and response to light exposure. (**a**) Schematic representation of the FTO/LFP NPs/DYE electrode; (**b**) open circuit voltage (OCV) under Neon light exposure (red line): the voltage after a plateau at 3.40 V increased to 3.75 V and in the dark using a black box (blue line), the voltage, as expected, slightly decreases from 3.44 to 3.41 V in 500 h. The inset shows the change in OCV upon illumination with a solar simulator (green line).

The original photocathode investigated consists of a film of colloidal LFP nanoplateletes^18^ deposited on conducting glass/F:SnO_2_(FTO), annealed and sensitized with the N719 dye as represented in Fig. 1a. Details of the film preparation are given in the ‘Methods' section.

**Figure 2:**
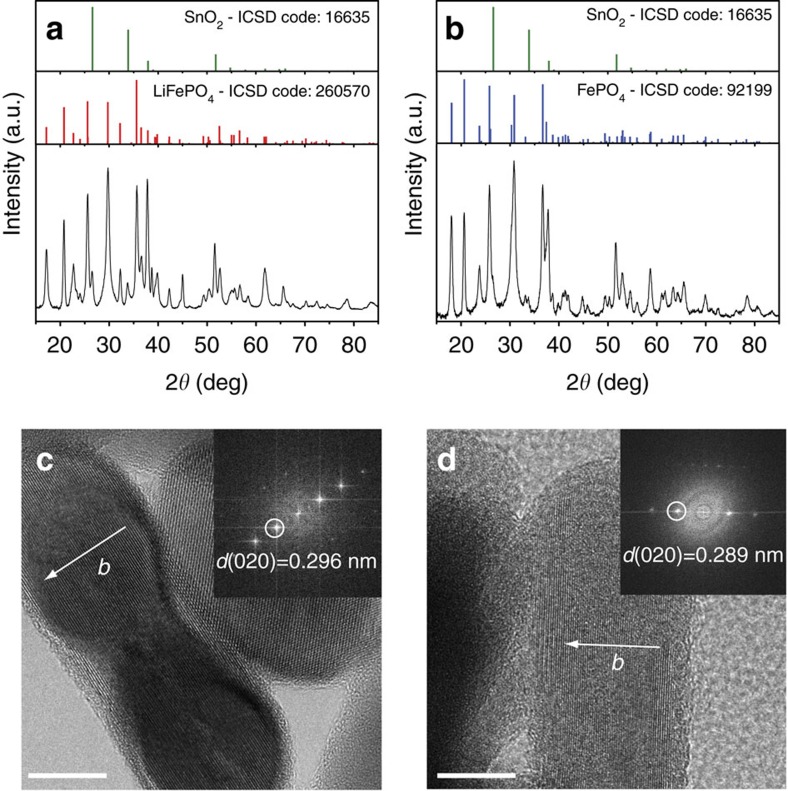
Characterization of LiFePO_4_ nanoplatelets before and after light exposure. (**a**) XRD pattern of pristine film of LFP, (**b**) XRD pattern of the film after light exposure, (**c**) HRTEM of pristine LFP (scale bars, 10 nm) and (**d**) HRTEM of LFP after light exposure (scale bars, 10 nm).

Subsequently, the ternary FTO–LFP–dye film was tested (Supplementary Figs 1 and 2) as the working electrode (WE), with lithium as the counter electrode (CE) and reference electrode in a solution of 1 M LiPF_6_ in EC/DEC (30/70 v/v+2% VC) as the electrolyte. The experiments were performed in a dry room under Neon ambient light (two Philips T8 32 W neon tubes, see the spectrum in Supplementary Fig. 3). The open circuit voltage (OCV) started at a plateau at 3.45 V and after 500 h increased to 3.75 V (red curve in Fig. 1b); upon replacing the Neon lamps with a solar simulator illumination (see ‘Methods' section) the same rise of OCV of LFP was much faster (∼30 h, see inset in Fig. 1b). We then performed the same OCV experiments in a black box where the voltage slowly dropped to 3.41 V (blue curve in Fig. 1b). The films, before and after OCV, were then analysed by X-ray diffraction (XRD). The presence of triphylite (LFP) in the pristine film was unambiguously highlighted by grazing incidence angle X-ray diffraction (GIXRD) measurement (Fig. 2a). After light exposure, the XRD pattern (Fig. 2b) shows the presence of heterosite (FePO_4_) only, suggesting the complete delithiation of the pristine triphylite phase by apparent photo-oxidation (no residual LFP was detected). Some extra peaks, assigned to cassiterite (SnO_2_), are present in both patterns due to the FTO layer in the substrate.

In contrast, the LFP phase is still preserved on the film after 500 h of OCV in the dark (Supplementary Fig. 4a). The film exposed to solar simulator light shows heterosite FePO_4_ while the film in the dark is LFP (Supplementary Fig. 4b). LFP nanocrystals, before and after OCV, were also analysed by high-resolution transmission electron microscopy (HRTEM). The images of Fig. 2c,d were acquired from [010] oriented crystals. The arrows mark the direction of the Li channels in the structure. A small difference of the lattice constant *b* is measured in the FFT transforms, in good agreement with the XRD patterns, and confirms the reduced volume of the delithiated structure (FePO_4_) with respect to the starting structure (LFP).

**Figure 3:**
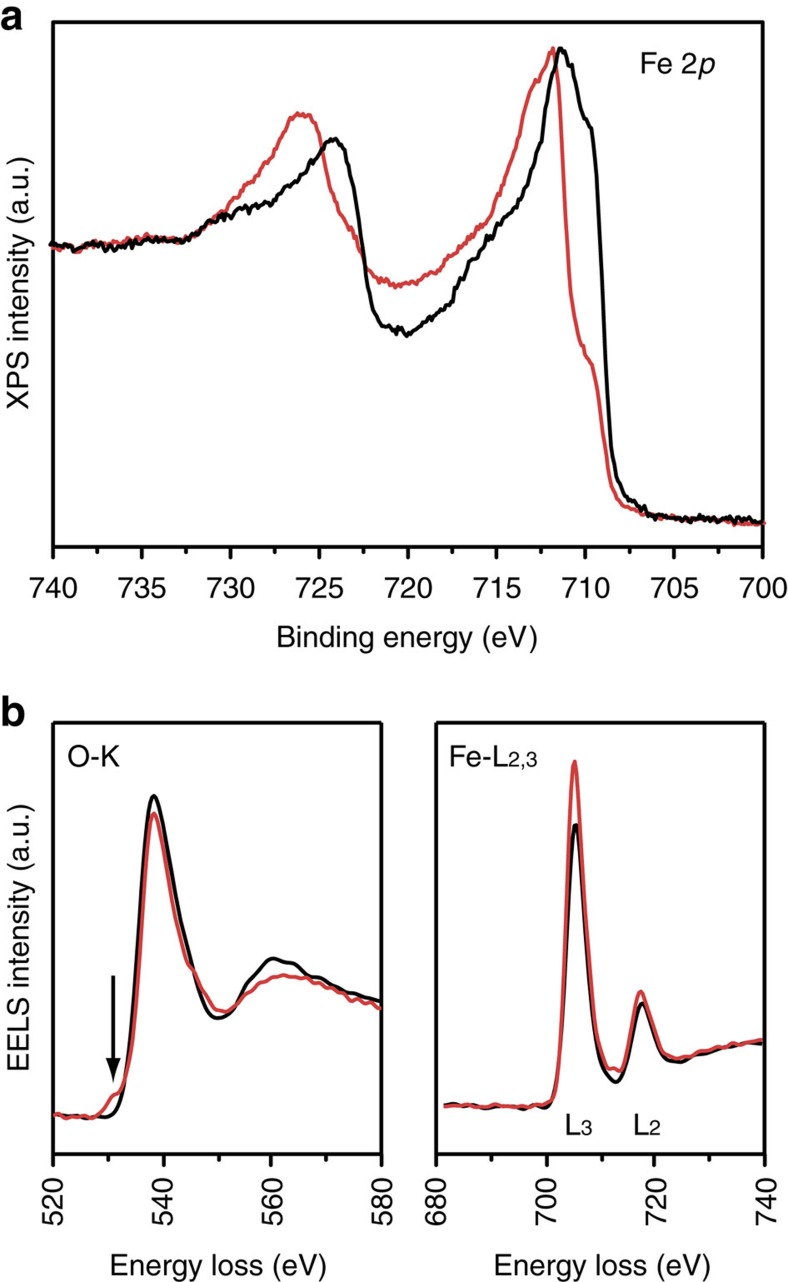
XPS and EELS analysis of LiFePO_4_ before and after light exposure. (**a**) Fe 2*p* XPS results of the FTO–LFP–dye film before (black line) and after (red line) light exposure. The data are shown after normalization and (**b**) EELS spectra of oxygen K edge and iron L_2,3_ edge before (black) and after (red) light exposure.

X-ray photoemission spectroscopy (XPS) was performed on the LFP sample before and after exposure to light, and the results obtained for the Fe 2*p* peaks are shown in Fig. 3a. The spectra collected on the sample before light exposure (black profile) resemble those obtained on the LFP nanoplatelets, as reported by Paolella *et al*.^19^, thus confirming that Fe is present as Fe(II) in the pristine material. In particular, the Fe 2*p* peaks are evident by their peculiar profile owing to multiplet splitting, also reported by Dedryvere *et al*.^20^. After light exposure (red profile), the low binding energy component centred at 709.7 eV decreased in intensity while the maximum of the Fe 2*p*_3/2_ peak shifted to a slightly higher binding energy (from 711.1 to 711.8 eV). A similar trend was already reported by Dedryvere *et al*.^20^ on cycled LFP electrodes and assigned to Fe^2+^ partial oxidation to Fe^3+^ during battery charging. In other words similar to standard battery charging, we have here photo-assisted oxidation of LFP. The quantitative analysis of the Fe(III)/Fe(II) ratio is complicated by the peculiar profile of both Fe(II) and Fe(III) components^20^, but we were able to assign the observed spectral changes to the delithiation of LFP nanoplatelets as a result of the photo-oxidation process.

Electron energy loss spectroscopy (EELS) showed the ionization edges of oxygen (O-K) and iron (Fe-L_2,3_) and verified the oxidation of Fe from Fe(II) to Fe(III) when delithiation occurs (Fig. 3b). A typical feature of oxidation with the formation of FePO_4_ is the pre-peak of the O-K edge^21^ as visible in the photo-oxidized sample. Moreover, the Fe-L_2,3_ should change correspondingly due to the different occupation of the Fe 3*d* bands. Indeed, the L_3_/L_2_ ratio (relative intensity of the two white-lines) increases in the photo-oxidized sample due to the higher amount of Fe(III) as expected^22^. The oxidation of Fe in this case does not involve the addition of oxygen atoms, as confirmed by the very similar integral intensity of the O-K spectra in the post-edge region (that is, same oxygen amount of atoms in the structure).

### Multiple LFP photo-oxidations

**Figure 4:**
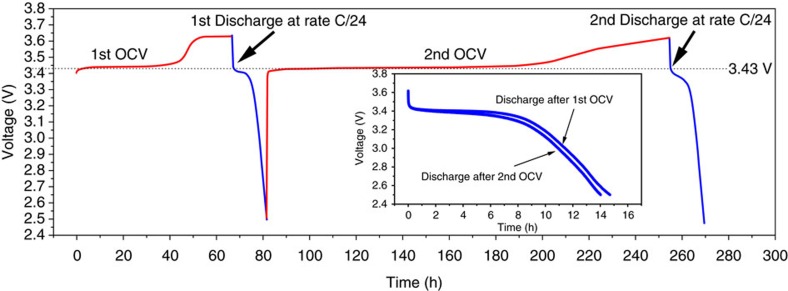
Open circuit voltage and discharge curves of LiFePO_4_**on FTO glass.** OCV charge (red lines) performed under solar simulator lighting and galvanostatic discharge (blue lines) at C/24.

The OCV was observed during exposure using a solar simulator (200 W lamp, see inset in Fig. 1 and Supplementary Fig. 4b). In this case, the full charge occurred faster (1.5 days versus 20 days) compared to the charge under neon light. Therefore, light is essential for the oxidation reaction. Also, the XRD measurements showed clearly the conversion of triphylite LFP into heterosite FePO_4_ after illumination by the solar simulator. The cell was subsequently subjected to OCV charging and discharging cycling (Fig. 4). As it can be seen after ∼70 h at OCV and charge, the battery reached 3.62 V and then discharged at C/24 (see ‘Methods' section for more details) to a capacity of 104 mAh g^−1^. The cell was held at OCV and charged a second time which required 100 h at OCV to reach 3.43 V and another 100 h to reach 3.62 V (increasing the voltage from 3.43 to 3.62 V needed 40 h more compared to the first OCV). After light-assisted charging, the cell was discharged a second time at C/24 where a comparable capacity of 99.3 mAh g^−1^ was obtained. The second experiment at OCV required more time probably due to partial dissolution of the dye in the electrolyte, but the reaction is still reversible. Using only LFP (Supplementary Fig. 5) we observed a capacity fading that is attributed to the absence of a binder in the LFP film, causing as result partial film delamination.

Subsequently an aliquot of the electrolyte was analysed by ^1^H and ^19^F NMR spectroscopy after cycling to verify its chemical stability. According to the NMR analysis (Supplementary Figs 6 and 7), the EC/DEC molar ratio slightly increases from 0.80 to 0.86 due to evaporation of DEC during the cell operation. More importantly, no new products are detected by ^1^H NMR. Unfortunately, the concentration of the N719 dye was too low to be detected if any part of it solubilized in the electrolyte. ^19^F NMR indicated that LiPF_6_ is the major component along with traces (<0.3%) of degradation products. One of these degradation product was POF(OH)_2_, similar to that identified by Wilken *et al*.^23^ and Campion *et al*.^24^ in Li-ion battery common electrolytes. Surprisingly, no traces of POF_3_, HF or LiF were observed with this sample, which are usually associated with the degradation reactions of LiPF_6_ resulting in the formation of POF(OH)_2_. Our NMR results therefore did not suggest any major changes taking place in electrolyte composition. However, as we discuss later in the mechanism section where we propose EC/DEC to react at the LFP/electrolyte interface that apparently went undetected by the NMR analysis (Supplementary Figs 24–27) due to the low concentrations involved.

**Figure 5:**
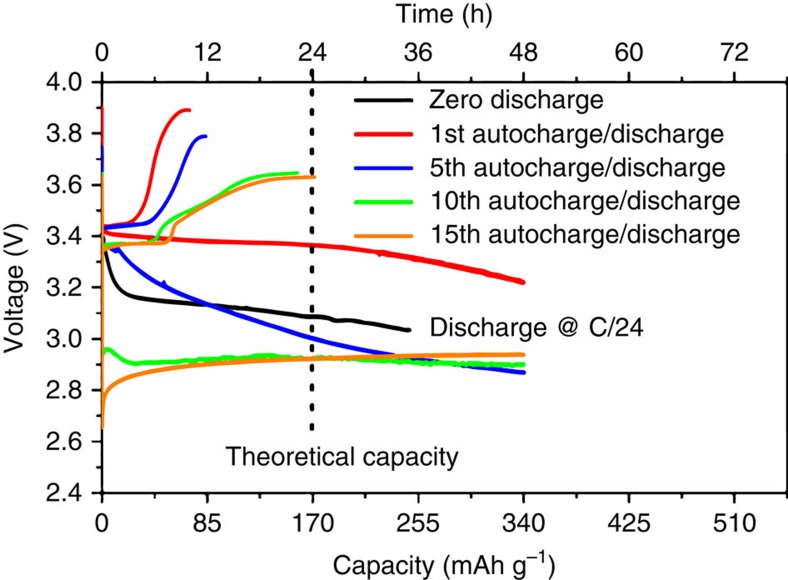
Open circuit voltage and discharge curves of LiFePO_4_ film on ITO. Open circuit voltage (OCV) curves and galvanostatic discharge profile of ITO@LFP+CNTs+PVDF. The 1st OCV/discharge curve is indicated with red line, the 5th one with a blue line, the 10th with green line while the 15th with orange line.

The stability of the film was improved by changing the composition of the film and its preparation (see ‘Methods' section). The changes made were the use of PET/Sn:In_2_O_3_ (PET/ITO) instead of glass/FTO (Supplementary Fig. 1) as substrate, the use of carbon nanotubes to improve conductivity (Supplementary Fig. 8), and the use of PVDF binder to improve film integrity. We performed XRD on the new film and obtained the same results as for glass/FTO: the conversion of triphylite into heterosite after OCV is shown in Supplementary Fig. 9. During galvanostatic charge–discharge performed in the dark (Supplementary Fig. 10), we observed a relatively low battery capacity/stability (below 40 mAh g^−1^). Nevertheless, Fig. 5 shows a relatively fast photo-assisted OCV (<24 h) and a discharge current for 48 h at C/24 corresponding to a capacity at least two times the theoretical one. The same capacity was observed after 15 discharges (and 15 OCVs photo-assisted charges) As mentioned earlier the photocathode tends to undergo partial charge during galvanostatic discharge because of its exposure to light that induces LFP to delithiate, hence creating vacancies for extra charge storage beyond the theoretical capacity. In Fig. 5, the 15th OCV is lower than the first OCV and probably due to a loss of dye in the electrolyte-although this could not be confirmed, but the photo-oxidation of LFP is still much faster with respect to the glass/FTO electrode (respectively 24 h after 15 cycles for ITO versus 160 h after the 2nd cycle for FTO). Considering these results we can reasonably assume that the combined use of PET/ITO, carbon nanotubes and PVDF as binder improved the performance of the device. Another critical factor in the observed photo-oxidation of LFP is the nanosize effect. This was determined by comparing the photo-response of differently prepared LFP samples. Thus no photo-oxidation was observed when we replaced the colloidal nanocrystals with hydrothermally synthesized crystals (∼1–3 μm sized crystals); but after ball-milling (crystals of ∼80 nm) photo-oxidation did take place as evidenced by the increase of OCV to 3.65 V after 24 h (for more details see Supplementary Fig. 12). BET analysis showed the pristine hydrothermal LFP sample has a specific surface area of 5 m^2^ g^−1^, while the ball milled sample has 24 m^2^ g^−1^. By comparison, the etched colloidal LFP sample (nanoplatelets 7–10 nm thick) with a surface area of ∼70 m^2^ g^−1^ underwent the fastest photo-oxidation. Therefore, the surface area of LFP nanocrystals is a key property that facilitates photo-oxidation. Our hybrid photocathode featuring colloidal LFP nanoplatelets and N719 dye can undergo a photo-oxidation for 15 cycles in a cell with Li metal as anode. Besides the cyclability, it is interesting to note that we observed (Supplementary Fig. 11), when the light was switched off, the voltage experienced a drop of 150 mV but when the light was switched on again, the voltage profile was restored. The voltage drop observed after 15 cycles (from 3.4 V to ∼3 V) is attributed to a number of factors as evidenced by supplementary characterization work, such as the formation of resistive LiF deposit on the surface of the film (Supplementary Fig. 13) and dye segregation at the surface of the cathode or its partial dissolution in the electrolyte (N719 was still detected in its active form by Raman analysis, see Supplementary Fig. 14).

Having demonstrated the photo-assisted oxidation of LFP in a two-electrode cell with Li metal as CE we proceeded to calculate the photo-conversion and storage efficiency (refer to Supplementary Fig. 15 and details in Supplementary Methods), which was found to be in the range 0.06–0.08%. Though these values are low we should bear that these are early performance data corresponding to electrodes made of LFP nanoparticles deposited on (transparent conductive oxide) TCO along C and a binder. Moreover hybrid devices made of solar cells and energy storage cells in tandem typically yield efficiencies <1% (ref. 25). The present system featuring a hybrid photo-cathode (LFP/dye) is unlike the previous in-series concept^9^ or three-electrode systems^25^. At this early stage we attribute such low current efficiency to large charge recombination losses at the LFP/dye/electrolyte interface, and this will require further interfacial engineering of electrodes.

### Charge transfer process

**Figure 6:**
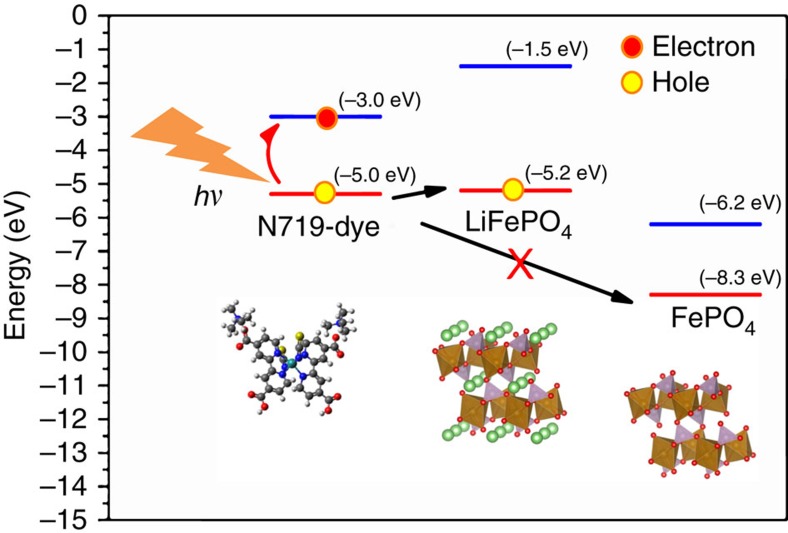
Energy band alignment of the photo-cathode components. The arrows illustrate the desired process: the absorption of photons excites the dye, that leads to hole injection into LiFePO_4_ particles; the injection of holes into the charged phase of FePO_4_ is forbidden.The work functions of LiFePO_4_ and FePO_4_ are calculated as reported in this work; the energy band of N719 is adapted from the work of Zhang *et al*.^66^.

Since LFP is converted to FePO_4_, there must be a charge transfer process involving electrons and/or holes that makes the LFP nanoparticles positively charged. Meanwhile, when the cathode is exposed to visible light, the only material that absorbs the photons is the dye, which has a HOMO–LUMO gap of 2.33 eV (green light), the charge transfer process must take place between the dye and the LFP particles as shown in Fig. 6.

To investigate this hypothesis, we performed first-principles calculations on bands position in LFP and FePO_4_, and aligned the energy bands of the relevant materials. This calculation is performed by determining the difference between the effective electronic potential in vacuum (*V*_vac_) and the valence band maximum (*E*_VBM_) of the material. The band offset relative to the vacuum level is *E*_off_=*V*_vac_–*E*_VBM_. The [010] surface of LFP exposed to vacuum was used in the calculation because this surface has the lowest surface energy, and consequently the highest equilibrium surface area^26^,^27^. We utilized the VASP package^28^ with the projector-augmented wavefunction scheme^29^,^30^ using the Perdew–Burke–Ernzerhof exchange correlation function^31^. The surface structure of LFP that is aligned perpendicular to the [010] direction plus 25, 30 and 35 Å-thick vacuum layers was relaxed until the forces on the atoms were <0.01 eV Å^−1^. The values reported here were obtained with the 35 Å vacuum layer, which converged the band positions very well. The thickness of LFP was five times greater than the dimension of the [010] direction of the lattice parameter in the calculation. Our test also shows that further increase in the LFP thickness does not significantly change the final result. The band offset of the maximum in the LFP valence band is −5.2 eV, as shown in Fig. 6. A calculation of the band offset of FePO_4_ by the same procedure yielded −8.3 eV. These aligned energy bands convinced us that the desired processes are as illustrated in Fig. 6. That is to say, the incident photon excites electrons in the dye molecule, pumping them to the excited LUMO, leaving a hole in the HOMO. When the LFP particles are positively charged by the holes, the electrochemical potential for Li^+^ increases. The equilibrium between the Li^+^ in the electrolyte and LFP particles shifts towards more Li^+^ in the electrolyte as the cathode is charged. However, when most of the LFP is transformed to FePO_4_, as illustrated in Fig. 6, the absorption of photons does not lead to hole transfer to FePO_4_, therefore the reaction stops. The undesired processes, such as charge carrier recombination, electrons hopping to FePO_4_ do exist and adversely affect energy conversion efficiency as already alluded earlier. Proper selection of cell components and interfacial electrode engineering should be pursued for improving the efficiency of the device. As for the electrons, it appears that they do not hop into FTO or ITO to any significant extent if consider that during OCV illumination no current was flowing between the two electrodes, lithium reduction is energetically unfavourable (see next paragraph) and the FTO/ITO collectors remained totally transparent, without showing any lithium intercalation product (that is, the TCO did not become brownish). The next section discusses the fate of photo-generated electrons.

### Fate of photogenerated electrons

Although the open circuit potential of the photo-cathode (WE versus Li_ref_) increases to 3.6–3.9 V, lithium ions cannot be reduced to lithium metal (an additional 1.5 eV is needed to complete the process). We therefore carried out a systematic analysis as to the fate of photo-excited electrons.

The photo-generated electrons did not reduce any crystalline component of the film as evident by XRD (Supplementary Fig. 2). LiF was observed at the surface of the film (Supplementary Fig. 13), but this is linked to electrolyte hydrolysis^32^ (as evidenced by the presence of hydrolysed electrolyte product POF(OH)_2_ and HF in the electrolyte, see NMR data in Supplementary Figs 24–27). Similarly, the dye could not have been reduced considering that its tiny amount (1:1,000) was able to oxidize the whole LFP for at least 15 times as confirmed by Raman analysis (Supplementary Fig. 14) that revealed the presence of N719 (refs 33, 34) in photo-oxidized sample with FePO_4_ (refs 35, 36). In addition no new impurities such as Li_2_O, Li_2_CO_3_ or LiOH were observed in the analysis of the film as confirmed by EELS analysis (Supplementary Fig. 16). Having ruled out the reduction of Li^+^ ions at the anode or the reduction of any component of the TCO/LFP/dye photocathode we propose that the photo-generated electrons react with some component(s) of the electrolyte used (LiPF_6_ in EC/DEC+VC). To verify this scenario, we tested different electrolytes: 1 M LiPF_6_ in EC/DEC+2% VC, 1 M LiPF_6_ in THF (in THF polymerization reactions are slower than in DME/DOL^37^), 1 M LiPF_6_ in TEGDME^38^, 1 M LiTFSI in EC/DEC +2% VC and 1 M LiTFSI in DME/DOL. We observed photo-oxidation of LFP to take place (after 24 h illumination) only when LiPF_6_ in EC–DEC+2% VC is used as electrolyte (Supplementary Fig. 17). This series of tests confirms that the photo-oxidation is facilitated by certain electrolyte components as are, for example, EC and DEC that are known to be prone to reaction^39^. We observed further that the reaction in LiPF_6_ in EC/DEC+2% VC involved oxygen as only in the presence of oxygen gas (dry room environment) and not in the presence of argon, did photo-oxidation take place (Supplementary Fig. 18). We propose the following reaction sequence to account for these observations: the photo-generated electrons reduce oxygen and the new reduced oxygen species (for example, peroxide and/or superoxide^40^) being unstable react with the carbonate-based electrolyte to form SEI as also has been observed in Li–O_2_ battery studies by Zhu^41^ and Read^42^. The formation of insoluble Li carbonate SEI species was confirmed indirectly by the data of Supplementary Fig. 21 that revealed the formation of cubic and polyhedral shaped organic Li carbonate crystals on the Li metal surface. However NMR could not detect any reduced electrolyte species (Supplementary Figs 24–27). The low reactivity of LiTFSI in EC/DEC+2% VC should be due to differences in viscosity and lithium ion solvation properties^43^,^44^ that hinder lithium release by LFP photo-oxidation.

We also found that when the LFP film was immersed in the LiPF_6_ in EC/DEC+2%VC electrolyte under light illumination without any lithium metal present, then no photo-oxidation was observed in 48 h. But photo-oxidation did take place after 7 days (Supplementary Fig. 19). These results suggest that the presence of lithium metal as CE accelerates the LFP photo-oxidation reaction via catalytic reduction reaction of the coupled oxygen/electrolyte. To verify the role of the lithium metal CE and the presumed reduction of the electrolyte (EC/DEC solvent components) via the reduced oxygen species formed by the photo-generated electrons, we performed a new OCV test under illumination using the two-electrode (LFP versus Li) cell configuration the results of which are given in Supplementary Fig. 20. As it can be seen the OCV of LFP increased with illumination time again and LFP converted to FePO_4_. Therefore, the presence of lithium, even without passage of electrons via the external circuit, is confirmed to facilitate this photo-oxidation reaction. We procedeed afterwards to examine the reaction products formed on the lithium metal (CE) surface after completion of the photo-oxidation test. The analysis revealed the formation of crystals (cubic and polyhedral; see Supplementary Fig. 21a,b) that contain lithium, carbon and oxygen as confirmed by energy dispersive X-ray spectrometry (EDS)^45^ (Supplementary Fig. 21c). However, no cubic or polyhedral crystals were observed to form after simple immersion of lithium metal for 48 h in 1 M LiPF6 EC/DEC +2% VC (Supplementary Figs 22 and 23) pointining to the link between photo-oxidation and SEI formation on Li anode. We have further performed EDS analysis on the Li metal anode after 48 h of discharge and we observed no more micron-sized cubic crystals that constituted the major part of the SEI but only rings composed of Li, C and O (Supplementary Figs 29–31). In other words, we observed just remnants of the SEI after discharge.

### Global mechanism of LFP photo-oxidation

**Figure 7:**
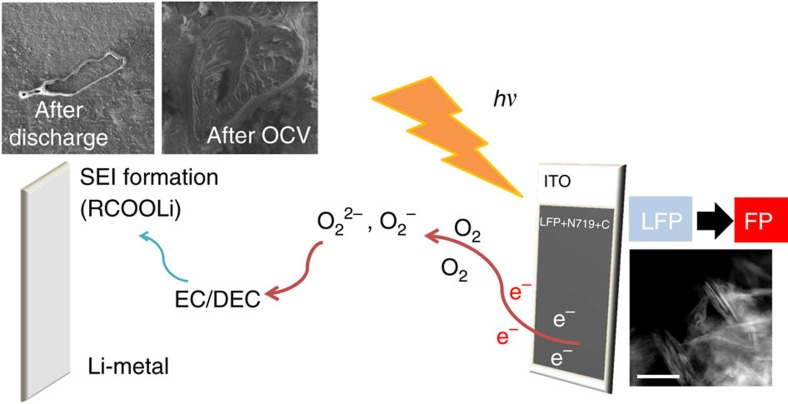
Global photo-assisted charging mechanism. LFP photo-oxidation by holes injected by the excited dye and formation of SEI via reduction of oxygen by photoelectrons in the LFP(dye)/electrolyte/Li cell. Organic carbonate-based electrolyte is decomposed by reaction with peroxide/superoxide generated by the photogenerated electrons and oxygen. The scale bar of TEM image is 200 nm.

Considering that no external current between the electrodes is possible during OCV illumination, that lithium is not deposited on the anode but rather consumed during the discharge, the composition of the electrolyte has an influence on the photo-oxidation reaction, that the presence of lithium metal facilitates the photo-oxidation of LFP, and that the SEI crystals formed on the lithium anode surface at least partially dissolves during discharge, we propose the following mechanism (as depicted in Fig. 7). The photo-assisted charging of LFP in the LFP(dye)/electrolyte/Li cell involves in addition to the oxidation of LFP via the injection of holes from the photo-excited dye, a two-step reaction sequence in which firstly the photo-generated electrons promote reduction of oxygen followed by the reaction with carbonate-based electrolyte (probably the ethylene carbonate^39^) and secondly lithium metal surface appear to provide favourable nucleation sites enabling the deposition of the new Li-carbonate-based electrolyte derivative components as Li compound crystal-containing SEI thanks to accumulated Li^+^ ions at the surface. The SEI film crystals mostly dissolve during discharging. The SEI does not appear to hinder the transfer of Li^+^ ions from the anode to the cathode during discharge. Therefore, the lithium metal facilitates the photo-oxidation reaction that mimics the charge process of a Li-ion battery under light irradiation, although no traceable Li reduction takes place at the anode. In this case the photo-generated electrons can be thought to be chemically stored as SEI at the lithium metal side.

In the present cell configuration, for energetic reasons mentioned earlier no new lithium metal is deposited at the anode during photo-assisted charging; this means that during the discharge lithium metal is consumed. In future work an alternative anode will have to be developed within an operating voltage of 0.7–1 V. In the meantime the high (∼double of the theoretical one) discharge capacities shown in Fig. 5 are due to continuing LFP photo-oxidation by N719 dye that keeps creating Li vacancies accommodating higher Li-ion storage than the stoichiometric formula suggests. This view is supported with the data presented in Supplementary Fig. 28 where LFP and FePO_4_ are seen to co-exist after 48 h of discharge under illumination.

**Figure 8:**
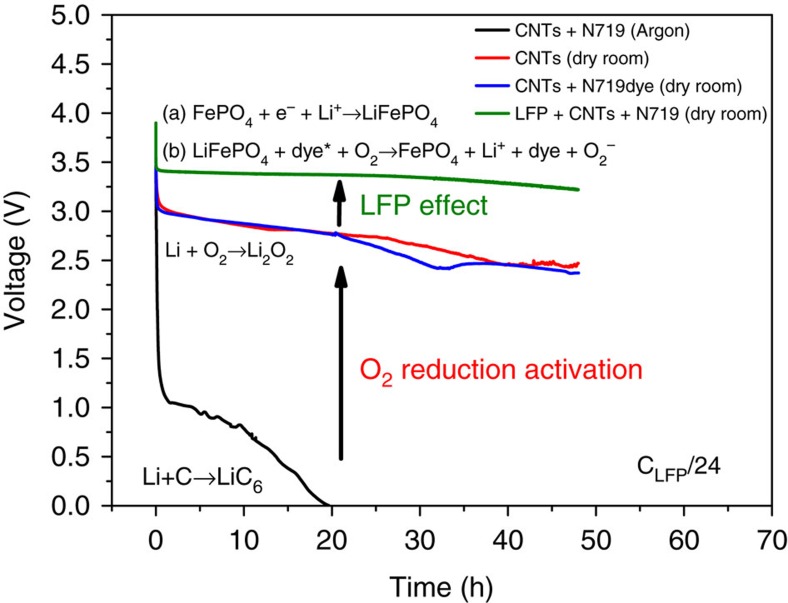
Discharge curves of films in different gas atmosphere at fixed C/24 discharge rate. Discharge curves of CNTs+N719 dye film under Argon gas (black line), CNTs+ N719 dye film in dry room (blue line), film of CNTs in dry room (red line) and LiFePO_4_+CNTs+dye film in dry room (green line).

To clarify the enabling role of oxygen we performed discharge tests of films using with and without LFP in either inert (argon) atmosphere or oxygen (dry room) atmosphere. The film tested were the standard LFP/CNTs/N19 film plus films of carbon nanotubes alone, or carbon nanotubes+N719 dye. The results are shown in Fig. 8. The two films of CNTs (with and without dye) tested in dry room showed a sloping voltage plateau starting at 2.9 V independent of the presence of the dye (with or without light exposure); by contrast no such plateau was observed in the case of the film tested in O_2_-free Ar-glove box^46^ indicating that the voltage plateau was associated with the reduction of O_2_ (refs 47, 48, 49). Carbon nanotubes are known to activate the reduction of O_2_ as suggested by Zhang^50^, Lim^51^ and Zelang^52^, but ruthenium^53^ in the diluted N719 dye plays a role only when LFP is present. In the light of these results, we can explain why the LFP/CNTs/N719 film shows high discharged (as mentioned above) capacities. Thus while FePO_4_ can be converted to LFP by the externally applied current during discharge, the formed LFP reconverts into FePO_4_ again by the photo-excited N719 dye that injects holes to LFP and electrons to oxygen, with the latter leading to formation of peroxide or superoxide species^40^,^54^,^55^,^56^ that subsequently react with carbonates.

## Discussion

In summary, we described the direct (open circuit) photo-oxidation of LFP nanocrystals by light irradiation in the presence of a N719-Ruthenium-dye as hybrid photo-cathode in a two-electrode system with Li metal anode and LiPF_6_-EC/DEC/VC electrolyte that corresponds to standard Li-ion battery charging. Dye-sensitization generates electron–hole pairs with the holes aiding the chemical conversion of high surface area LFP (triphylite) nanoplatelets to FePO_4_ (heterosite) at the cathode and electrons utilized in the formation of SEI at the anode via oxygen reduction. LFP (∼3.4 V versus Li^+^/Li), in analogy with the I_3_^−^/I^−^ couple (∼3.1 V versus Li^+^/Li) in DSSCs, acts effectively as a reversible redox agent for the regeneration of the dye N719. Photo-oxidation of LFP is pronounced with colloidal nanoplatelets but less so with hydrothermally synthesized crystals reflecting the strong nanosize/surface area effect. The SEI consists of organic lithium carbonate deposits-their formation of which is driven by the reduction of oxygen gas into per-/super-oxide species followed by the reaction of the latter with carbonate electrolyte components and accumulated of Li ions at the surface of the Li metal anode. Upon discharge most of the SEI dissolves and is reconstituted partially with repeated photo-charging and galvanostatic discharging cycling. The generated discharge current corresponds to a capacity at least two times the theoretical value of LFP. The same capacity was observed after 15 discharges (and 15 OCV photo-assisted charges). The excess capacity is attributed to the fact that the photocathode continues undergoing partial charge during galvanostatic discharge because of its exposure to light that induces LFP to delithiate, hence creating vacancies for extra charge storage.

The combined photo-conversion and storage efficiency of the prototype two-electrode cell with LFP/CNTs/N719 as photo-cathode and Li metal as CE was calculated to be in the range 0.06–0.08%. Though these values are low we should keep in mind that these are early performance data corresponding to electrodes made of LFP nanoparticles deposited on (transparent conductive oxide) TCO along carbon and a binder. To put this into perspective hybrid devices made of tandems of solar cells and energy storage cells yield till very recently efficiencies <1% (ref. 25). Moreover, the present system featuring a hybrid photo-cathode (LFP/dye) is unlike the previous in-series concept^9^ or three-electrode systems^25^. At this early stage, we attribute such low current efficiency to large charge recombination losses at the LFP/dye/electrolyte interface. Our findings open new possibilities in designing photo-rechargeable Li-ion batteries based on a two-electrode device configuration. Among the critical issues that need to be tackled before such an exciting nanotechnology device becomes a reality include interfacial engineering of the LFP/dye photo-cathode to reduce charge recombination losses and use of a reversible redox mediator that can accept the photo-generated electrons thus suppressing undesirable electrolyte reduction reactions. Also it is important to select an anode that can provide an operating voltage of ∼0.7–1 V as suggested by redox flow battery systems^57^,^58^,^59^,^60^,^61^,^62^ and iodine based systems^13^,^63^,^64^.

## Methods

### Materials

Lithium iodide (beads, ≥99%), lithium hydroxide, iron(II) chloride anhydrous (≥98%), iron sulphate heptahydrate (≥99.0%), lithium hydroxide monohydrate (≥98.0%), phosphoric acid (85% w/w in water, ≥99.9% trace metals basis), ammonium phosphate dibasic (≥98%), ammonium hydroxide (solution 28.0–30.0% NH_3_ basis), ascorbic acid (≥99.0%), oleylamine (>70%), 1-octadecene (>90%), ethanol and dichloromethane, Di-tetrabutylammonium *cis*-bis(isothiocyanato)bis(2,2′-bipyridyl-4,4′-dicarboxylato)ruthenium(II) dye (N719 dye, 95%), tetrahydrofuran (THF, anhydrous, ≥99,9%), tetraethylene glycol dimethyl ether (TEGDME, ≥99.0%), *N*-methyl pyrrolidone (NMP, >97%), vinylene carbonate (VC) (≥97%) and bis(trifluoromethane) sulfonimide lithium (LiTFSI) salt were purchased from Sigma Aldrich while carbon nanotubes were purchased from CNano Ltd. Polyvinylidene fluoride (PVDF) 7305 binder was purchased from Kureha (Japan). The chemicals were used without further purification. FTO was purchased from Cytodiagnostics Inc. Lithium metal was purchased from FMC lithium. 1 M LiPF_6_ in EC/DEC (3/7 +2%VC), 1 M LiTFSI in DME/DOL and EC/DEC solution were provided by BASF.

### Synthesis of colloidal LFP

LFP was synthesized by a colloidal route^18^. In a typical colloidal synthesis test, 2.25 g (16.8 mmol) lithium iodide (LiI), 1.65 g (12.5 mmol) dibasic ammonium phosphate, 1.575 g (12.5 mmol) iron(II) chloride, 125 ml (0.38 mmol) oleylamine and 125 ml 1-octadecene were mixed in a 500 ml three-neck flask connected to a standard Schlenk line. The solution was kept under vacuum at 120 °C for 1 h, after which it was heated to 250 °C under N_2_ for at least 3 h. The suspension was then cleaned by repeated additions of dichloromethane and ethanol followed by centrifugation at 8,000 r.p.m.

### Etching treatment

The LFS nanocrystals were subjected to etching via LiPF_6_ treatment to remove residual oleylamine ligand^19^. The colloidal LFP nanoparticles (400 mg) were dispersed in 10 ml of chloroform, and 500 mg of LiPF_6_ was dissolved in 10 ml of water. The two solutions were mixed and the final 20 ml mixture was vigorously shaken. After a few minutes, the LFP nanoplatelets were transferred into the aqueous phase and then collected and centrifuged at 8,000 r.p.m. Ultrapure water (40 ml) (resistivity of 18 MΩ cm^−1^) was added to the powder to remove the excess LiPF_6_ from the NCs. The NCs were then redispersed in 5 ml ultrapure water. This last step was repeated three times. A total of 5.5 g of etched LFP was prepared by this method.

### Synthesis of hydrothermal LFP

In a standard hydrothermal synthesis (see previous publications for more details^65^) 33.6 g (0.12 mol) of FeSO_4_ 7H_2_O, 15.41 g (0.36 mol) of LiOH H_2_O, 13.83 g (0.12 mol) of H_3_PO_4_, 0.5 g of ascorbic acid (C_6_H_8_O_6_) are mixed with 300 ml of deionised water in a glass liner. The final molar ratio between Li: Fe:PO_4_:C_6_H_8_O_6_ was 3:1:1:0.008. The pH was controlled at 7.8 by drop-by-drop addition of ammonium hydroxide NH_4_OH. The synthesis is performed in a stirred autoclave (OM-JAPAN). The sample was collected after 5 h at 180 °C. The ball milled hydrothermal LFP was obtained by ball milling SPEX for 30 min.

### Brunauer–Emmett–Teller (BET) measurements.

Specific surface area measurements were carried out by nitrogen physisorption at 77 K in a Quantachrome equipment, model autosorb iQ. The specific surface areas were calculated using the multi-point BET (Brunauer–Emmett–Teller) model, considering 11 equally spaced points in the *P*/*P*_0_ range from 0.05 to 0.35. Prior to measurements, samples (50–200 mg in form of powder) were degassed for 1 h at 30 °C under vacuum to eliminate weakly adsorbed species.

### FTO—film preparation

A total of 5.5 g LFP NCs were dispersed in 57.5 ml deionized water under vigorous stirring for 24 h, then 0.4 ml Triton X100 was added to the solution and stirred for at least 48 h. The suspension was vigorous stirred for 48 h before coating. The film was prepared by dip coating using an RDC-15 dip-coater from Bungard. These films were obtained after three dip steps, with 20 s intervals between each step. The films were pulled up at a rate of 5 cm min^−1^ between the two immersions and dried for 4 min and dipped again. Then the film was annealed under nitrogen at 400 °C and cooled at room temperature in a VBF-1200X oven (MTI Corporation). Before the annealing step, the oven was vacuum purged three times and under N_2_ flow for 1 h, and the substrates were placed in a graphite crucible to avoid oxidation of LFP during annealing. The films have a thickness of ∼2.0 (±0.2) μm (Supplementary Fig. 2). The density of the LFP film is 1.43 mg cm^−2^ that corresponds to 59% porosity.

### FTO—electrode preparation

The electrodes were prepared from FTO/LFP films that were dipped in 10^−4^ M ethanol solution containing Ru-dye N719 for 24 h. Then the sample was dried under vacuum at 50 °C for 24 h.

### ITO—film preparation

A total of 5.5 g of LFP, 0.30 g of carbon nanotubes, 0.005 g of N719 dye and 0.30 g of PVDF 7305 were mixed with SPEX mixer adjusting the viscosity of the slurry with NMP solvent.The final ratio of LFP:CNTs:PVDF was 90:5:5. The amount of dye N719 was fixed to 5 mg for 5.5 g of LFP and it was added directly to the slurry during mixing with the SPEX. The viscosity of the slurry was adjusted using *N-*methyl pyrrolidone. The final film was prepared on PET/Sn:In_2_O_3_ (PET/ITO) and laminated. The CNTs sample was prepared mixing 5 g of CNTs and 0.5 g of PVDF adjusting the viscosity with NMP. The slurry was deposited on ITO film by doctor blade. The CNTs+N719 sample was prepared adding to the mixture 0.005 g of N719 dye.

### Electrochemical measurements

The electrochemical experiments were performed in a dry room with a three-electrode cell using metallic lithium as reference electrode and CE, and LFP on FTO/glass as WE. The electrolyte was 1 M LiPF_6_ in EC/DEC (3/7)+2% VC. The OCV data were recorded using a VMP3 potentiostat from Biologic. The OCV and galvanostatic charge/discharge measurements were conducted under light exposure and in the dark. About experiment under Ar atmosphere, the three-electrode cell was prepared in Ar-filled glove box and sealed with glue. The OCV measurement was performed in dry room.

### X-ray diffraction

GIXRD analysis was performed on a PANalytical Empyrean X-ray diffractometer equipped with a 1.8 kW Cu K_α_ ceramic X-ray tube, operating at 45 kV and 40 mA. The diffraction patterns were collected at room temperature, with incident angle *α* of 1.3° and a 2*θ* angular range of 15–85°, with a step size of 0.04°. A flat pyrolytic graphite monochromator was used to suppress the Cu K_β_ radiation and X-ray fluorescence. XRD patterns shown in Supplementary Figs 12, 19, 20 and 28 were recorded on a Rigaku SmartLab X-Ray diffractometer equipped with a 9 kW Cu K_α_ rotating anode (operating at 40 kV and 150 mA) and D/teX Ultra 1D detector set in X-ray reduction mode. The diffraction patterns were collected at room temperature in Bragg–Brentano geometry over an angular range 2*θ*=15–85° with a step size of 0.025°.

### Transmission electron microscopy

HRTEM images were acquired on a JEOL JEM-2200FS microscope at 200 kV. A 20 eV slit (Ω filter) was used to filter the elastic scattered electron to increase image contrast. EELS was acquired using the filter in spectroscopy mode, with a choice of apertures and convergence angle to provide identical sample acquisition conditions. The spectra were background subtracted and normalized in the post-edge regions of the Fe L_2,3_ spectrum to account for the thickness differences between the samples. The spectra were finally aligned at the energy onsets of O-K (∼532 eV) and Fe-L_2,3_ (∼708 eV) edges.

### High-resolution scanning electron microscopy

HRSEM observation shown in Supplementary Fig. 8 was carried out using a JEOL JSM-7500FA scanning electron microscope, equipped with a cold field emission gun (single crystal tungsten <310> emitter, ultimate resolution of 1 nm) and operating at 15 kV.

A dual-beam high-resolution microscopy from TESCAN (Czech Republic) was also used for observation (Supplementary Figs 21–23, 29 and 30) and local chemical analysis using a windowless energy dispersive spectrometer coupled with very low electronic noise from Oxford Instrument (see below EDS analysis).

### X-ray photoelectron spectroscopy

XPS was performed on a Kratos Axis Ultra DLD spectrometer using a monochromatic Al K_α_ source (15 kV, 20 mA). Wide scans were acquired at analyzer pass energy of 160 eV. High-resolution narrow scans were performed at constant pass energy of 10 eV and steps of 0.1 eV. The photoelectrons were detected at a take-off angle Φ=0° with respect to the surface normal. The pressure in the analysis chamber was maintained below 7 × 10^−9^ Torr for data acquisition. The data were converted to VAMAS format and processed using Casa XPS software, version 2.3.16. The binding energy (BE) scale was internally referenced to the C 1*s* peak (BE for C–C=284.8 eV).

### Nuclear magnetic resonance spectroscopy

^1^H and ^19^F NMR spectra were recorded on a Brucker Avance III spectrometer at 300 and 282 MHz, respectively. The following abbreviations were used for multiplicity assignments: ‘s' for singlet, ‘d' for doublet, ‘t' for triplet, ‘m' for multiplet, and ‘br' for broad. Deutered acetonitrile (CD_3_CN) was used as the reference solvent (dilution factor=5). Spectra were referenced to the solvent peak in ^1^H NMR, while the chemical shift of Li-PF_6_ was set to –76.9 p.p.m., in ^19^F NMR, as reported by Wilken *et al*.^23^.

### Electrolyte tests

We tested five different electrolytes: 1 M LiPF_6_ in EC/DEC+2% VC, 1 M LiPF_6_ in THF, 1 M LiPF_6_ in TEGDME, 1 M LiTFSI in EC/DEC+2% VC and 1 M LiTFSI in DME/DOL. LiPF_6_ in EC/DEC+2% VC and LiTFSI in DME/DOL are commercial products (see ‘Materials'). LiTFSI (1 M) in EC/DEC+2% VC, 1 M LiPF_6_ in THF, 1 M LiPF_6_ in TEGDME (ref. 38) are not commercial products and then were prepared separately. LiPF_6_ was dissolved in THF and TEGDME; no LiPF_6_ in DME/DOL could be prepared due to DME (and DME/DOL) polymerization by LiPF_6_ reactions^37^.

### Raman spectroscopy

The Raman measurement was conducted on a LabRaman Aramis Spectrometer (Horiba, Jobin Yvon). A laser beam of wave length 532.1 nm and energy ∼1.3 mW was focused with a × 50 objective. The measurements were conducted between 100 and 1900 cm^−1^ with data collection time of 2 min.

### Energy dispersive X-ray spectrometry analysis of lithium species

EDS analysis was performed using a newly develop very low energy EDS from Oxford instruments (UK). This detector is using a very low noise and optimized electronics together with removal of any window to decrease the absorption of very low energy X-rays in front of the detector crystal. This detector is unique to Hydro-Quebec and it is the result of a joint-collaboration between Oxford Instruments (UK) and H.Q (Canada)^45^.

### Solar simulator

The solar simulator was purchased from Sciencetech Inc, Model: (SLB-300B) Compact Solar Simulator Class ABA with Air Mass AM1.5 G Filter as a standard testing condition. Supplementary Figure 3 in our supporting information was obtained by measuring the light sources in our lab with a monochromator coupled with a pyroelectric detector. Calibration was done with a reference cell (a photodetector which is a monocrystalline silicon solar cell of dimensions 1 cm × 1 cm) purchased and calibrated from PV Measurements, Inc., Model: RC1-G5. When the reference cell's short-circuit current output equals its calibrated value of short-circuit current, this indicates that the irradiance reaching the reference cell is equivalent to the irradiance (one-sun) that was present during its calibration. When the reference cell's short-circuit current output equals its calibrated value of short-circuit current, this indicates that the illumination on the reference cell is equivalent to the calibrated standard one sun illumination (∼100 mW cm^−2^). With this method, we have determined the one-sun working distance and then placed our photo-battery set-up for the measurement.

### Data availability

The data that support the findings of this study are available from the corresponding author on request.

## Additional information

**How to cite this article:** Paolella, A. *et al*. Light-assisted delithiation of lithium iron phosphate nanocrystals towards photo-rechargeable lithium ion batteries. *Nat. Commun.* **8,** 14643 doi: 10.1038/ncomms14643 (2017).

**Publisher's note**: Springer Nature remains neutral with regard to jurisdictional claims in published maps and institutional affiliations.

## Supplementary Material

Supplementary Information Supplementary Figures, Supplementary Methods and Supplementary References
