# Supplementary material for: Light-assisted delithiation of lithium iron phosphate nanocrystals towards photo-rechargeable lithium ion batteries
Source: Nat Commun. 2017 Apr 10;8:14643. doi: 10.1038/ncomms14643 (PMC5394232; doi:10.1038/ncomms14643)
Supplement: Supplementary Information — Supplementary Figures, Supplementary Methods and Supplementary References [file ncomms14643-s1.pdf]

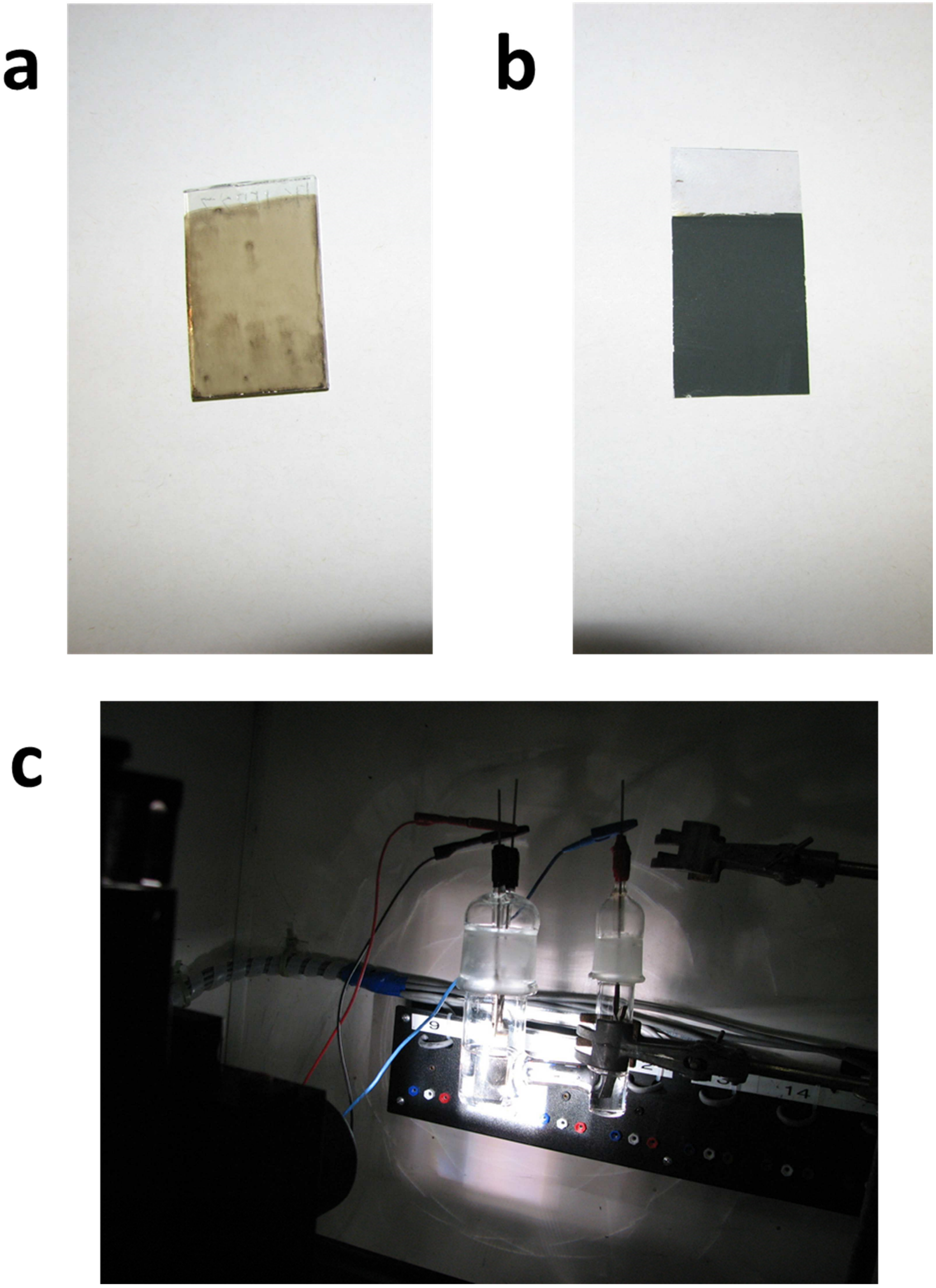

Supplementary Figure 1 Picture of films and setup used: Picture of (**a**) LFP film on FTO and (**b**) LFP/CNTs/N719/PVDF on ITO while the image in (**c**) represents the three electrode cell under light exposure in dry room

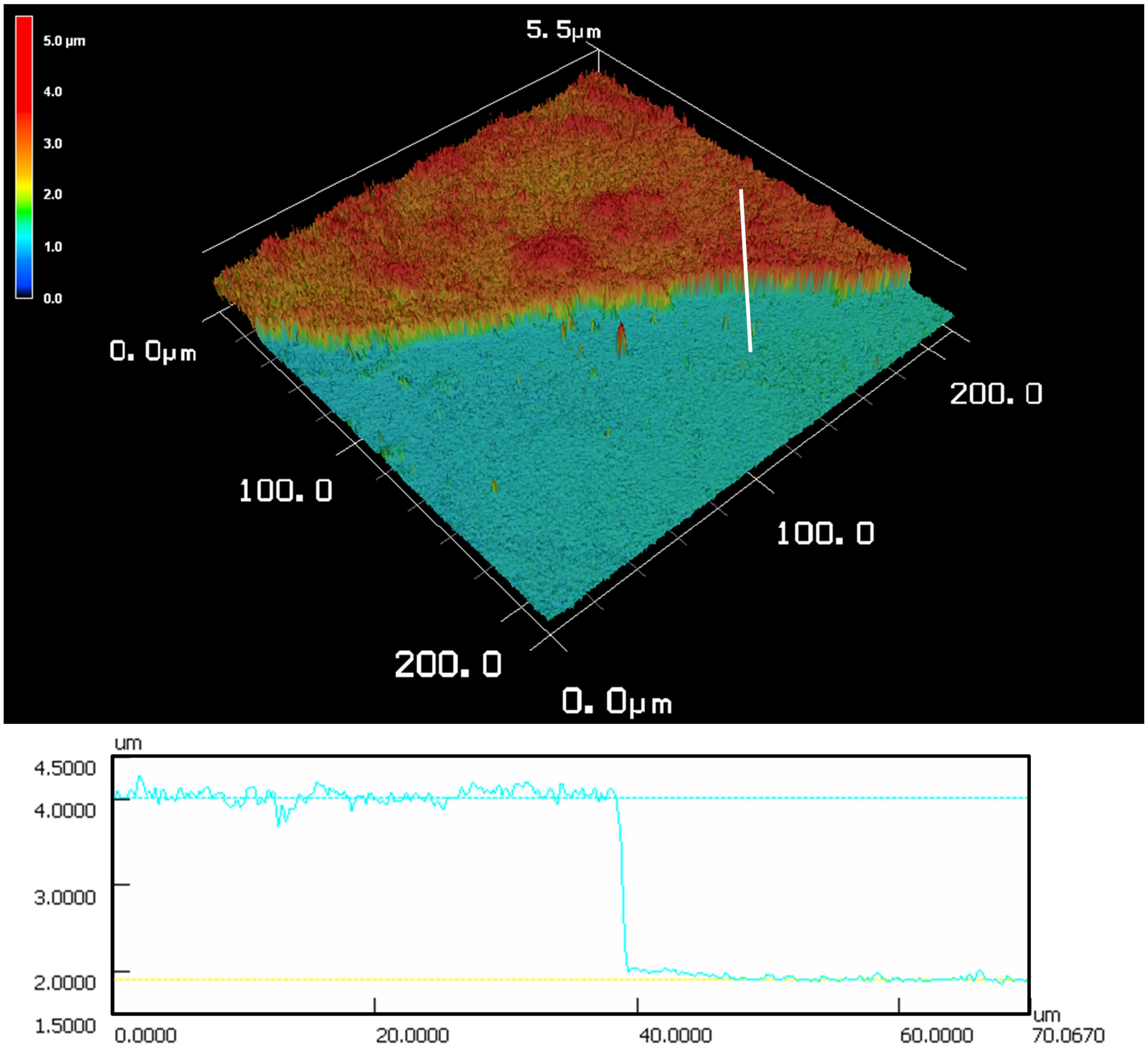

Supplementary Figure 2 Profile of the film: Thickness profile of $LiFePO_4$ FTO film obtained with a Keyence VK-X200 optical profilometer. The step was obtained by masking one part of the sample during dipping with Kapton tape ®.

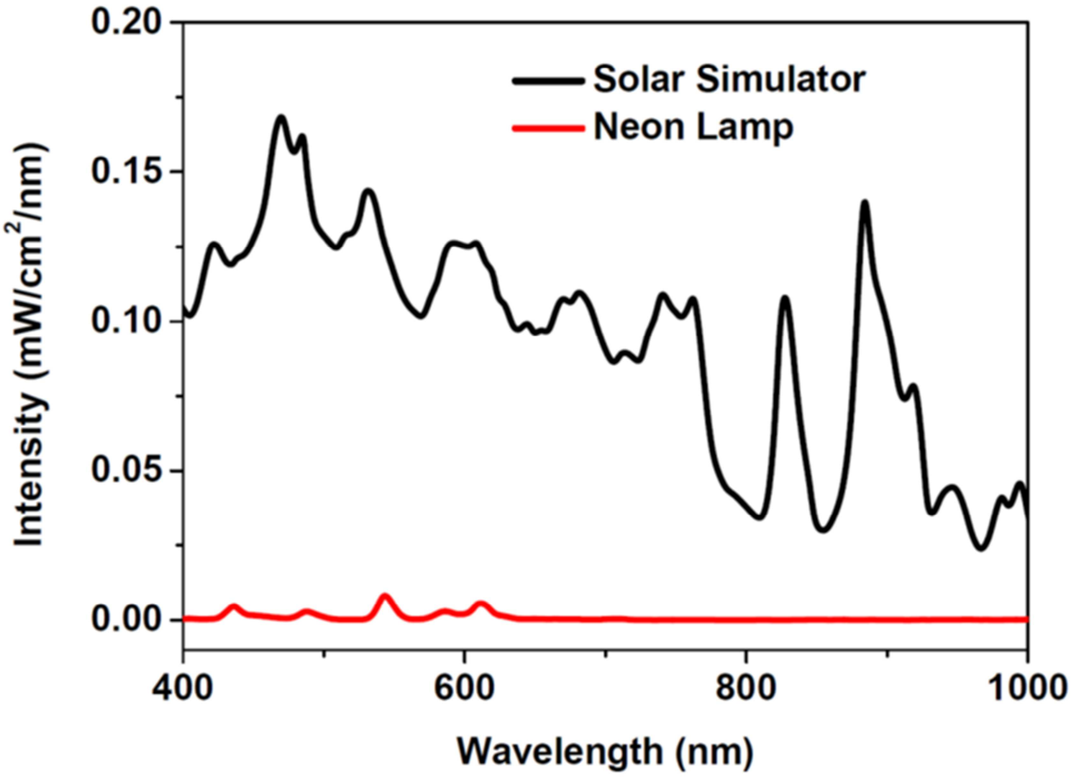

Supplementary Figure 3 Irradiance: Irradiance spectra of the solar simulator (black line) and Neon lamp (red line)

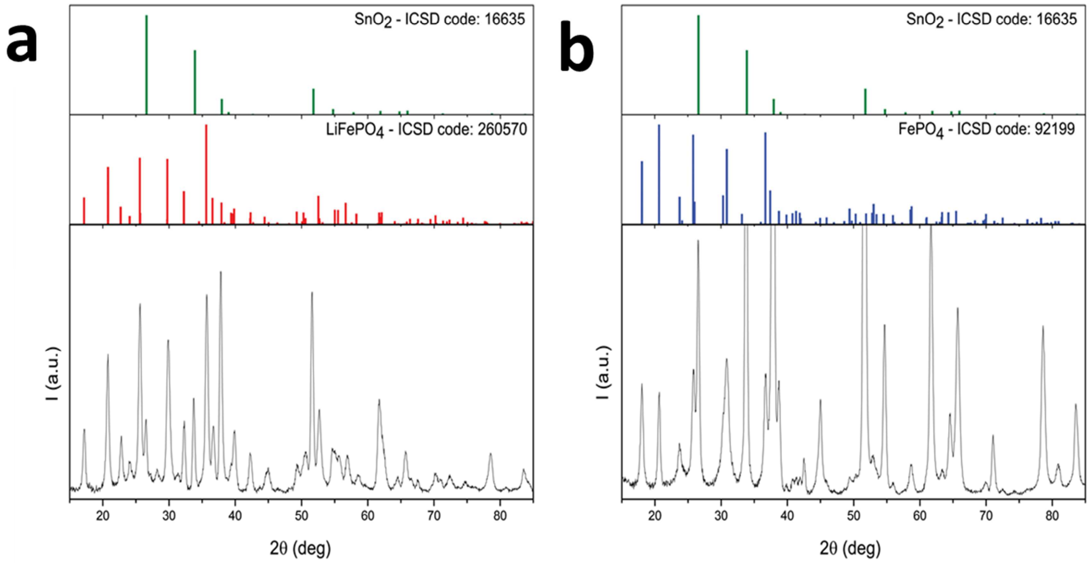

Supplementary Figure 4 XRD patterns of the FTO film before and after photo-oxidation under solar simulator irradiation: XRD patterns of (**a**) $LiFePO_4$ cathode after OCV in the dark and (**b**) after OCV with solar simulator

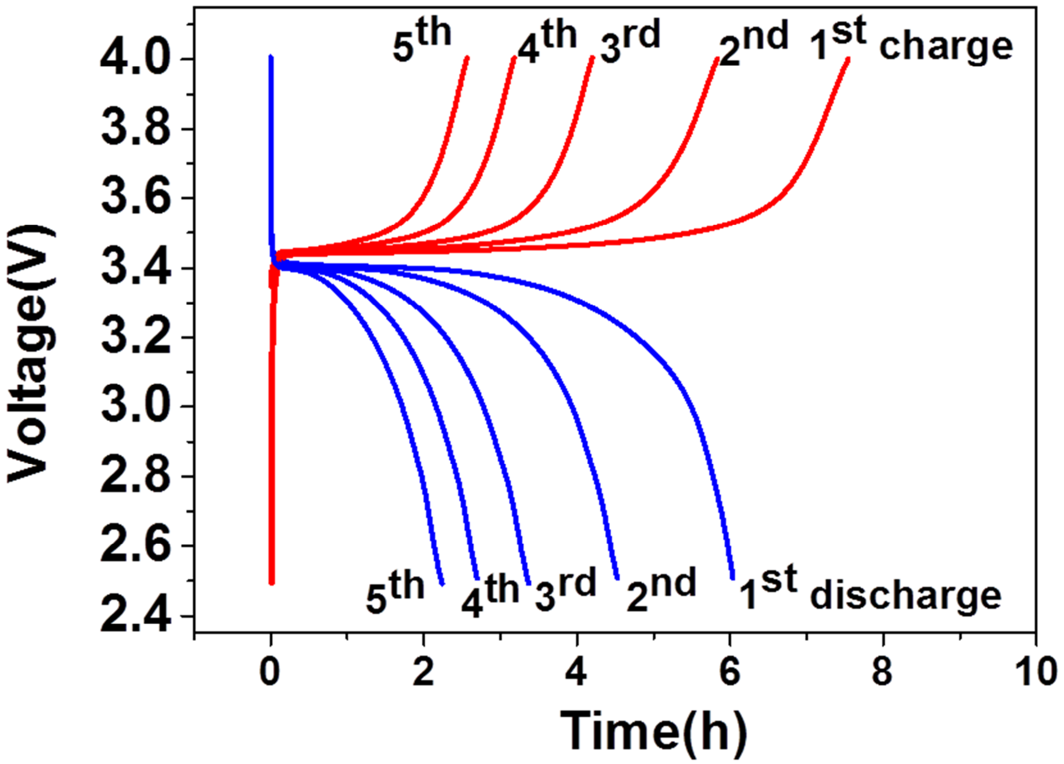

Supplementary Figure 5 Cycling curves of the film without N719 dye:
Charge/discharge profiles at C/24 for LFP film (without any N719 dye addition)

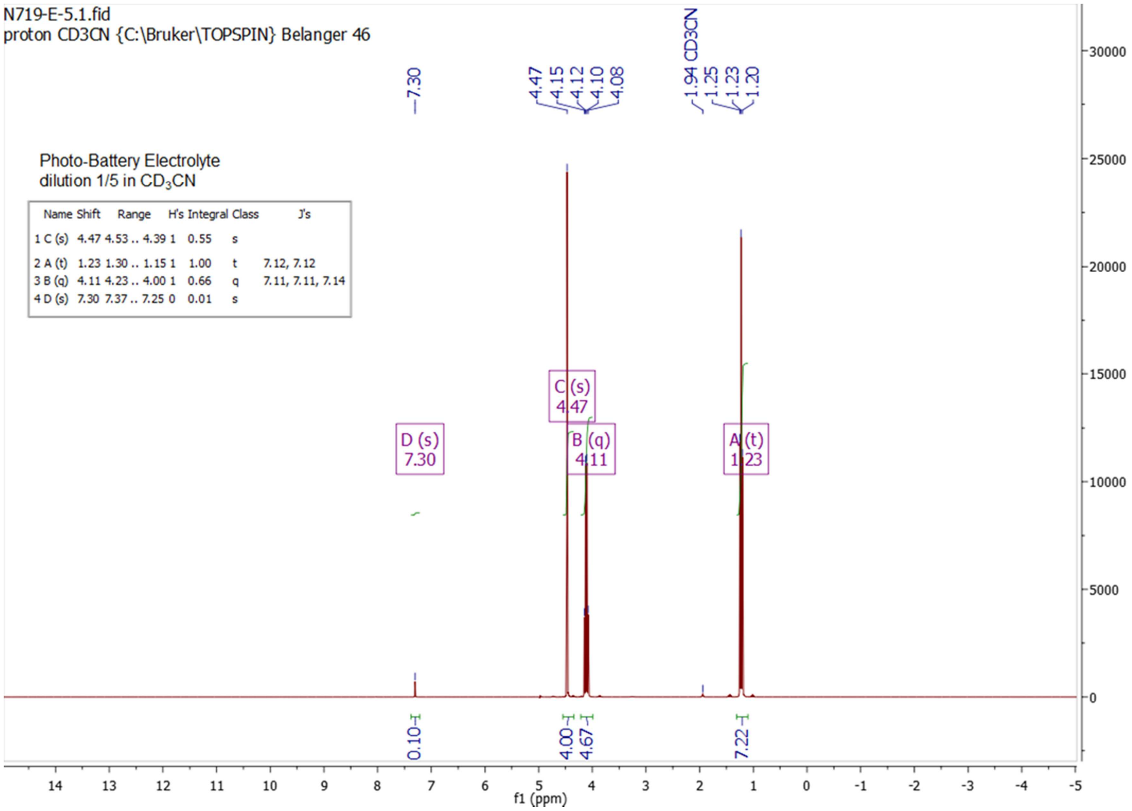

Supplementary Figure 6 Proton NMR analysis of the electrolyte after cycling: ^{1}H NMR spectra of an aliquot of the electrolyte after three cycles diluted by a factor five in deuterated acetonitrile (CD_3CN). Spectra were compared with those of a fresh electrolyte solution 1M $LiPF_6$ in EC/DEC (3/7) + 10% VC and compounds were identified according to their chemical shift and compared to results obtained in the literature[1-2]. The peaks were assigned as it follows: ^{1}H NMR (300 MHz, CD_3CN) δ (ppm): 1.23 (t, J = 7.1 Hz, 7.2H, CH_3 in DEC); 4.11 (q, J = 7.1 Hz, 4.7 H, CH_2 in DEC); 4.47 (s, 4H, CH_2 in EC); 7.30 (s, 0.1H, CH in VC).

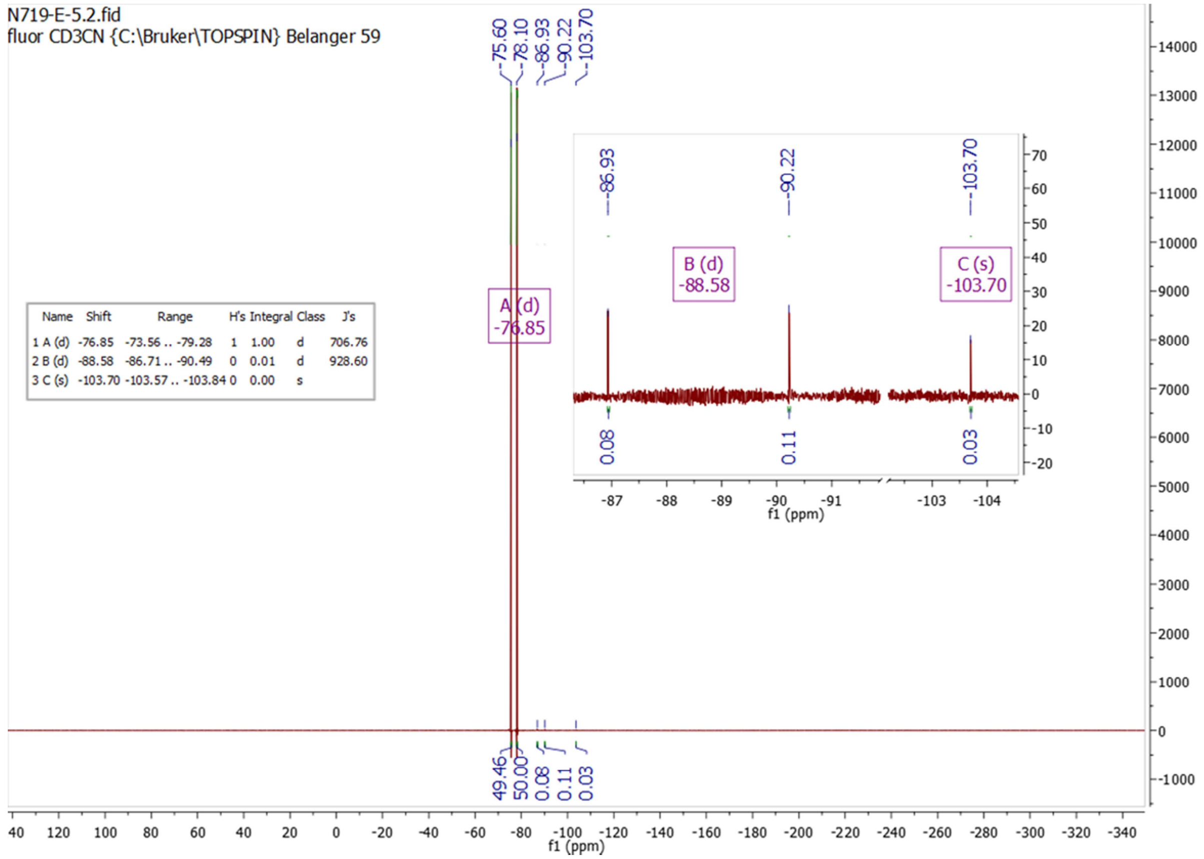

Supplementary Figure 7 Fluorine NMR analysis of the electrolyte after cycling: ^{19}F NMR spectra of an aliquot of the electrolyte after three cycles diluted by a factor five in deuterated acetonitrile (CD_3CN). Spectra was compared with those of a fresh electrolyte solution 1M $LiPF_6$ in EC/DEC (3/7) + 2% VC and compounds were identified according to their chemical shift and compared to results obtained in the literature[1-2]. The peaks were assigned as it follows: ^{19}F NMR (282 MHz, CD_3CN) δ (ppm): -103.7 (s, unknown); -88.6 (d, 929 Hz, POF(OH)2); -76.9 (d, 707 Hz, PF6-).

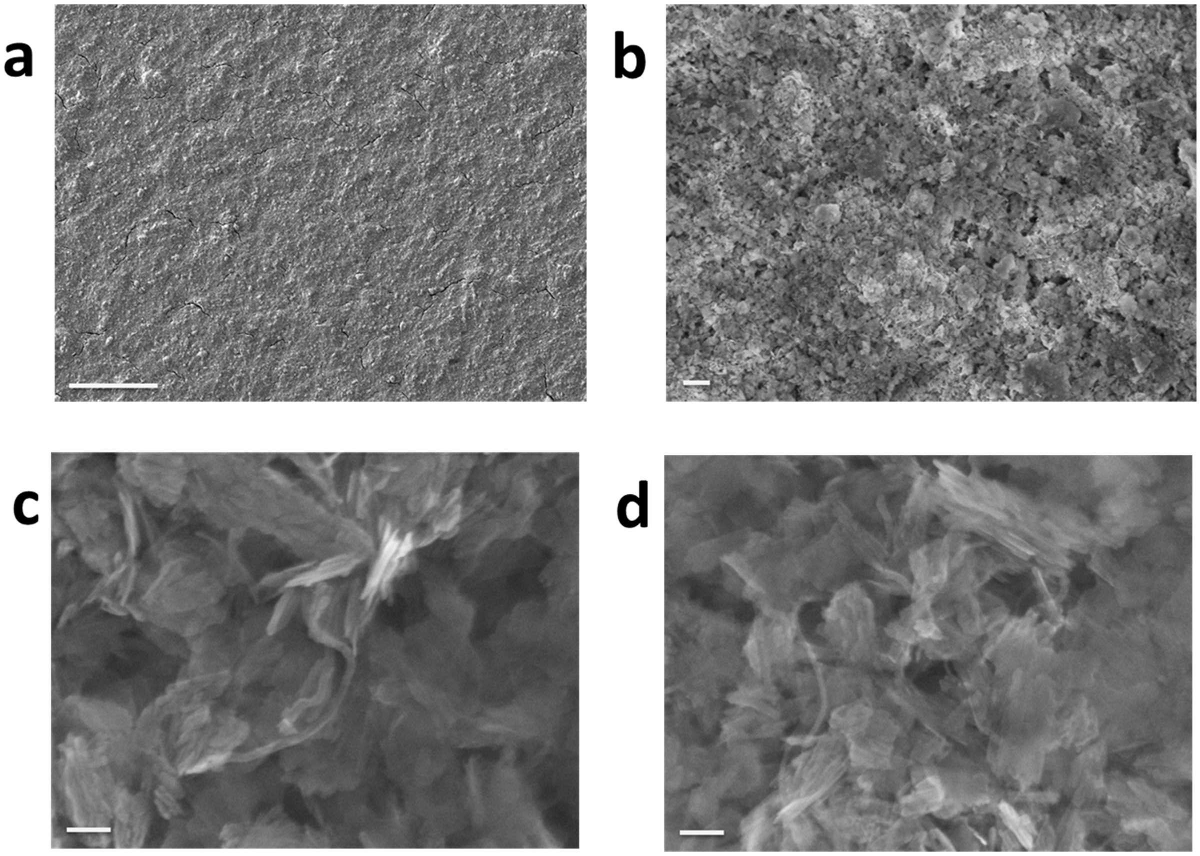

Supplementary Figure 8 SEM image of the film: Scanning electron microscopic (SEM) images of $LiFePO_4$+N719 dye +carbon nanotubes (CNTs) film coated on ITO substrate (scale bars respectively 100 µm, 1 µm and100nm).

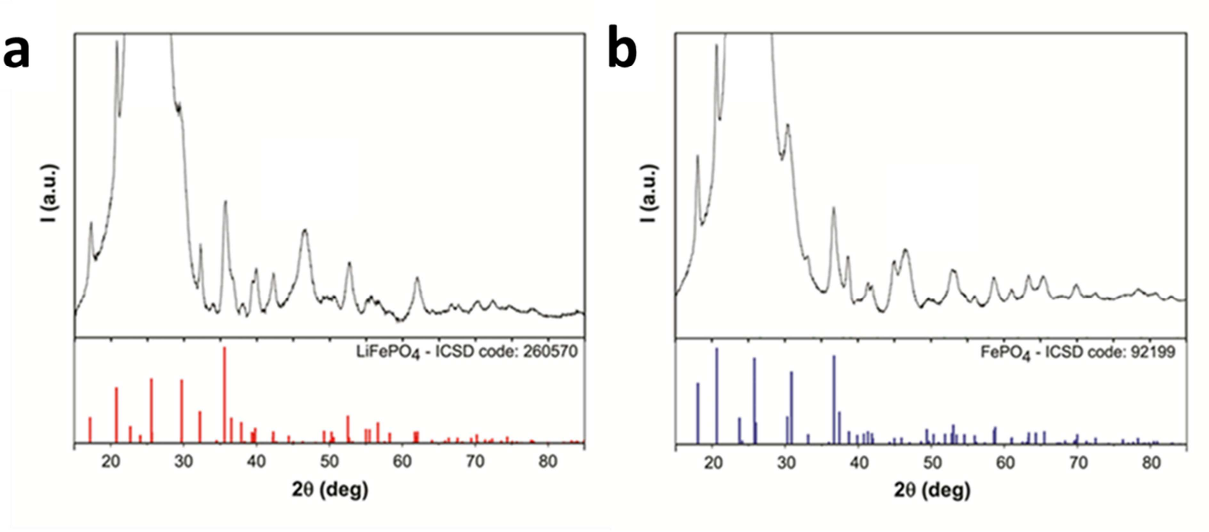

Supplementary Figure 9 XRD patterns of the ITO film before and after photo-oxidation : (**a**) XRD pattern before photo-oxidation and (**b**) after photo-oxidation of $LiFePO_4$+-N719+CNST film deposited on PET/ITO

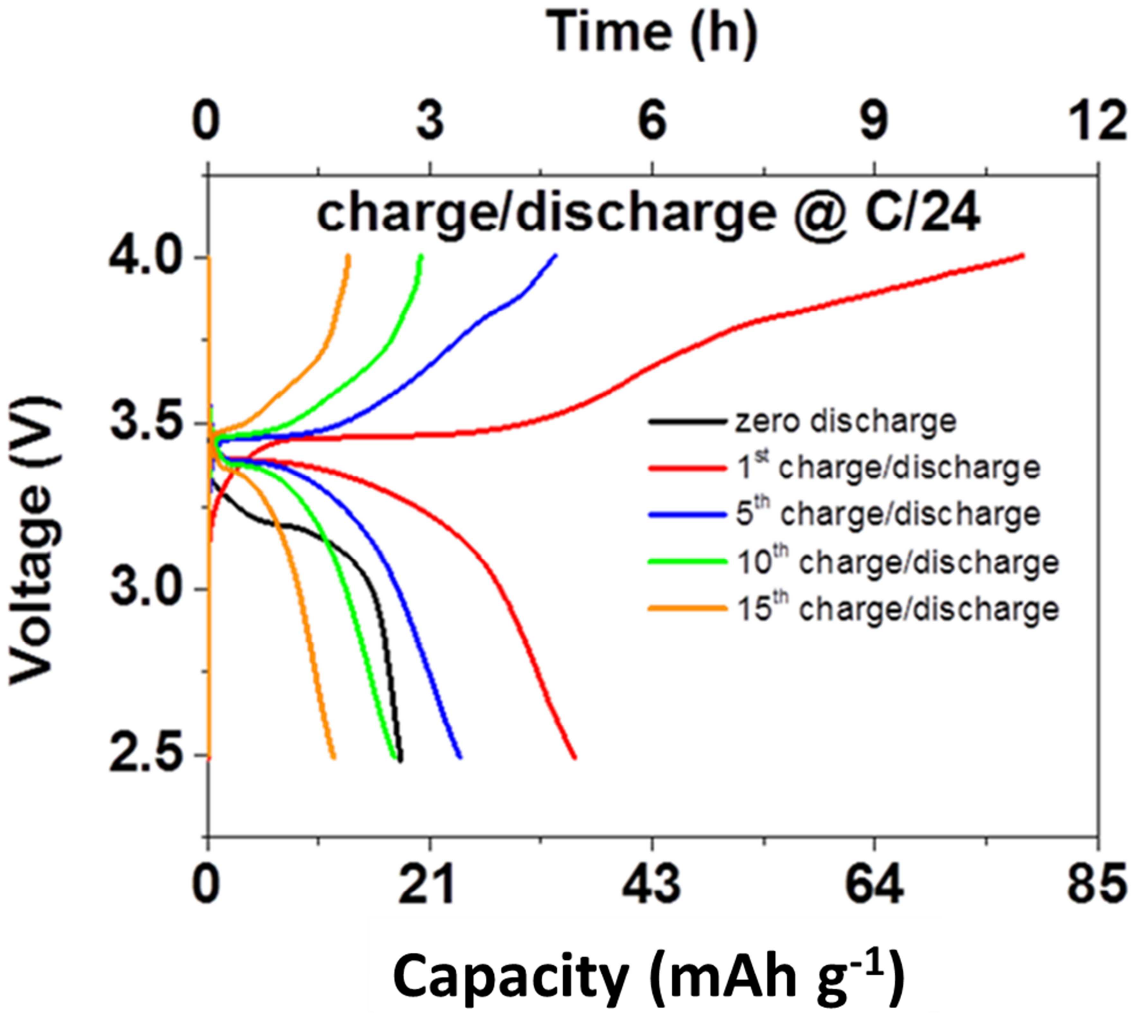

Supplementary Figure 10 Cycling curves of the ITO film: Galvanostatic charge/discharge at C/24 in dark of $LiFePO_4$+N719+CNT film deposited on PET/ITO

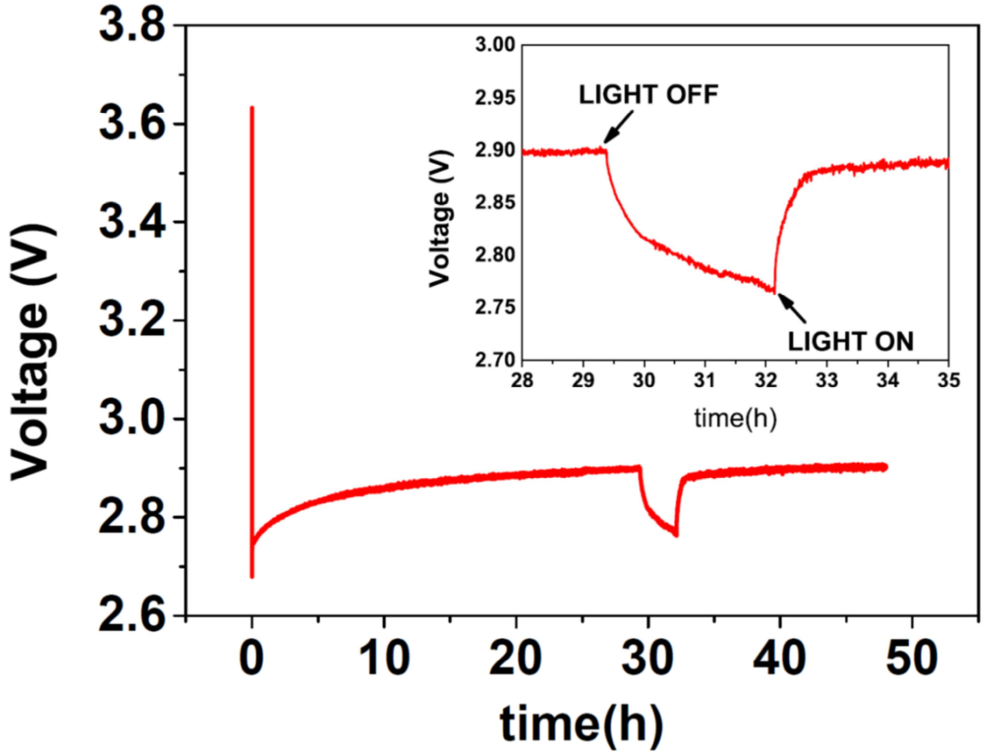

Supplementary Figure 11 Light response of the ITO film: 14th discharge at C/24 of $LiFePO_4$-N719 dye +CNT film deposited on PET/ITO. Inset is zoomed area of potential vs.time curve at which the sun light illumination is switched off and on during discharge

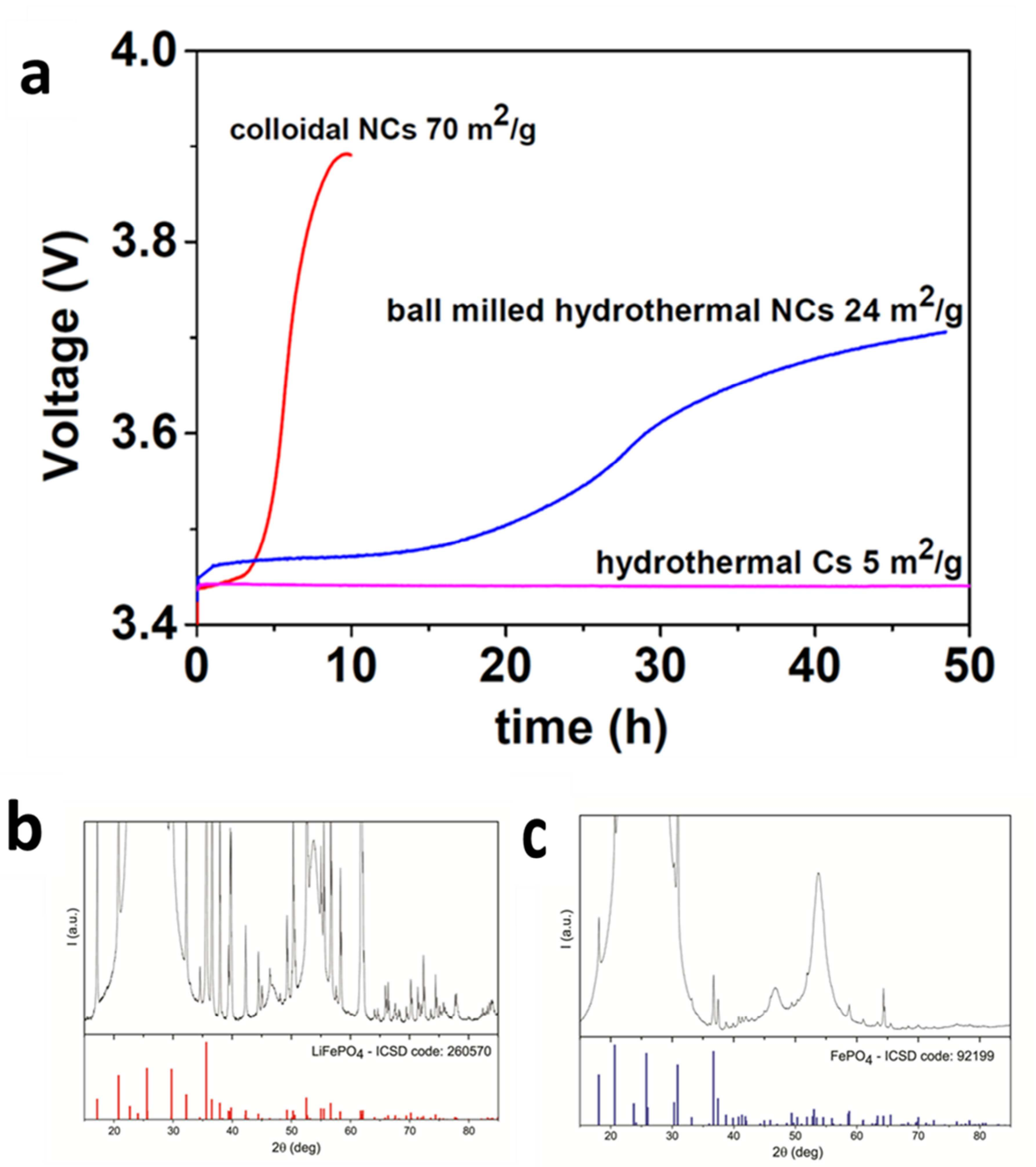

Supplementary Figure 12 LFP size effect dependence of photo-oxidation: (**a**) Open circuit voltage of different $LiFePO_4$ crystals under illumination: colloidal (red curve), ball milled hydrothermal (blue curve) and hydrothermal (magenta), (**b**) XRD pattern of hydrothermal $LiFePO_4$ crystals after OCV illumination, and, (**c**) XRD of ball milled hydrothermal $LiFePO_4$ crystals after OCV illumination

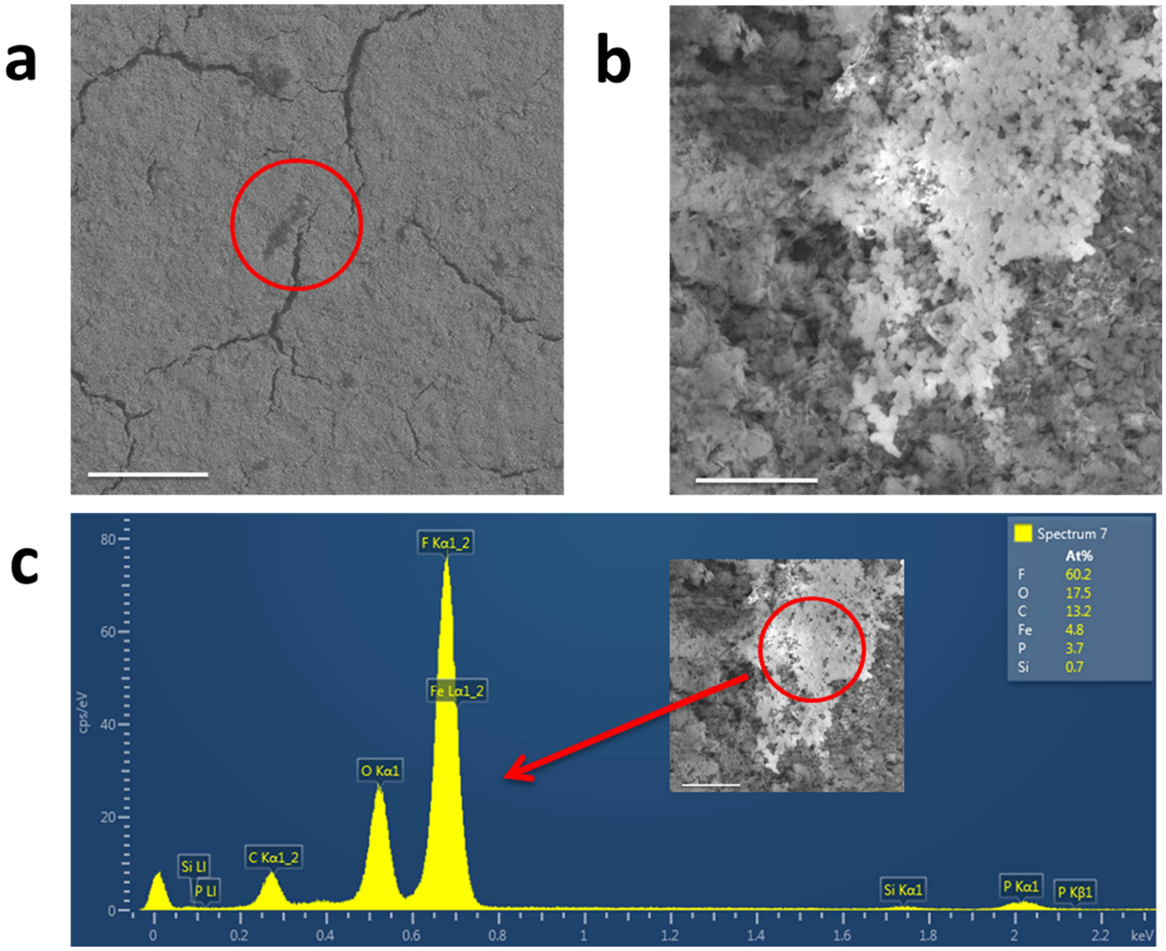

Supplementary Figure 13 SEM image and X-ray analysis of the surface: (**a**) (scale bar 50 µm) & (**b**) (scale bar 2 µm) SEM images and (**c**) Local X-ray analysis spectrum of the photo-oxidized cathode revealing the presence of LiF.

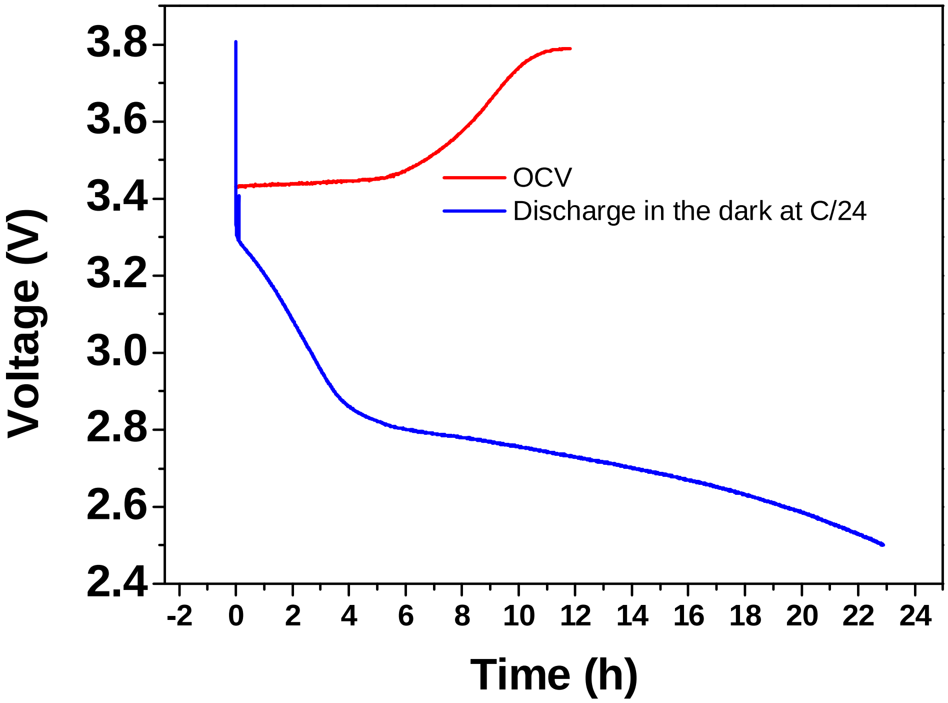

Supplementary Figure 14 OCV and discharge in the dark of the ITO film: OCV (red line) and discharge curve in the dark at C/24 rate (blue line)

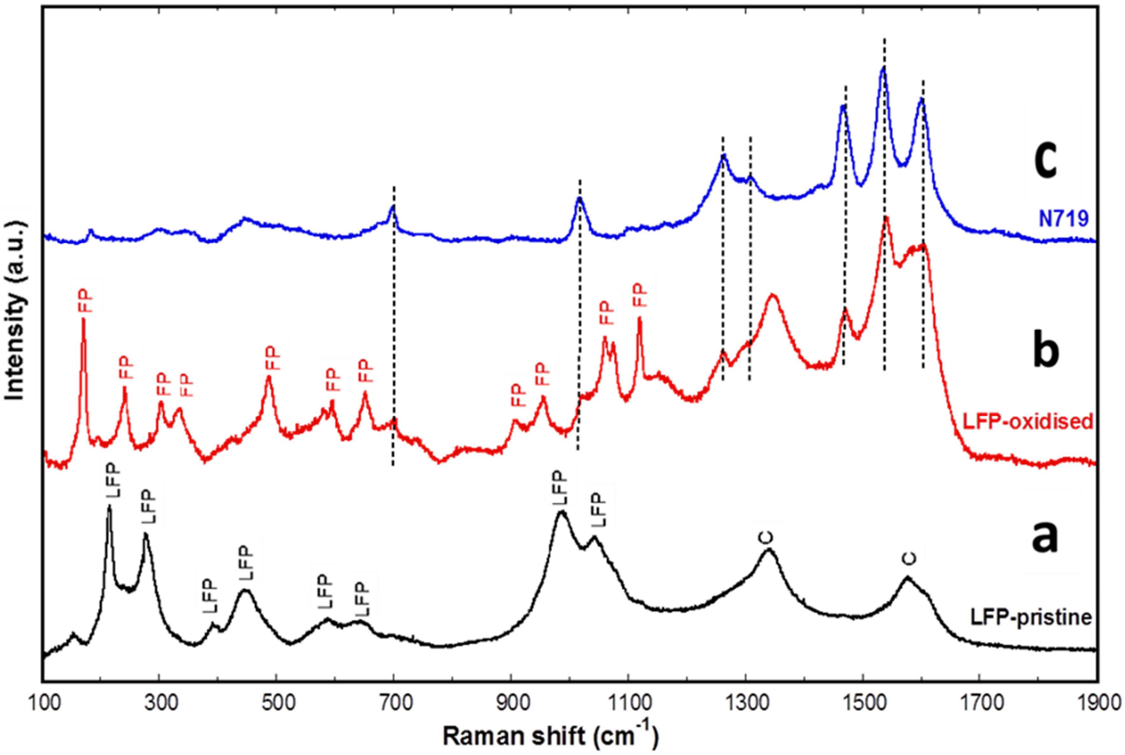

Supplementary Figure 15 Raman analysis: Raman spectra of (**a**) LFP as made electrode; (**b**) photo-oxidized electrode; (**c**) N719 dye.

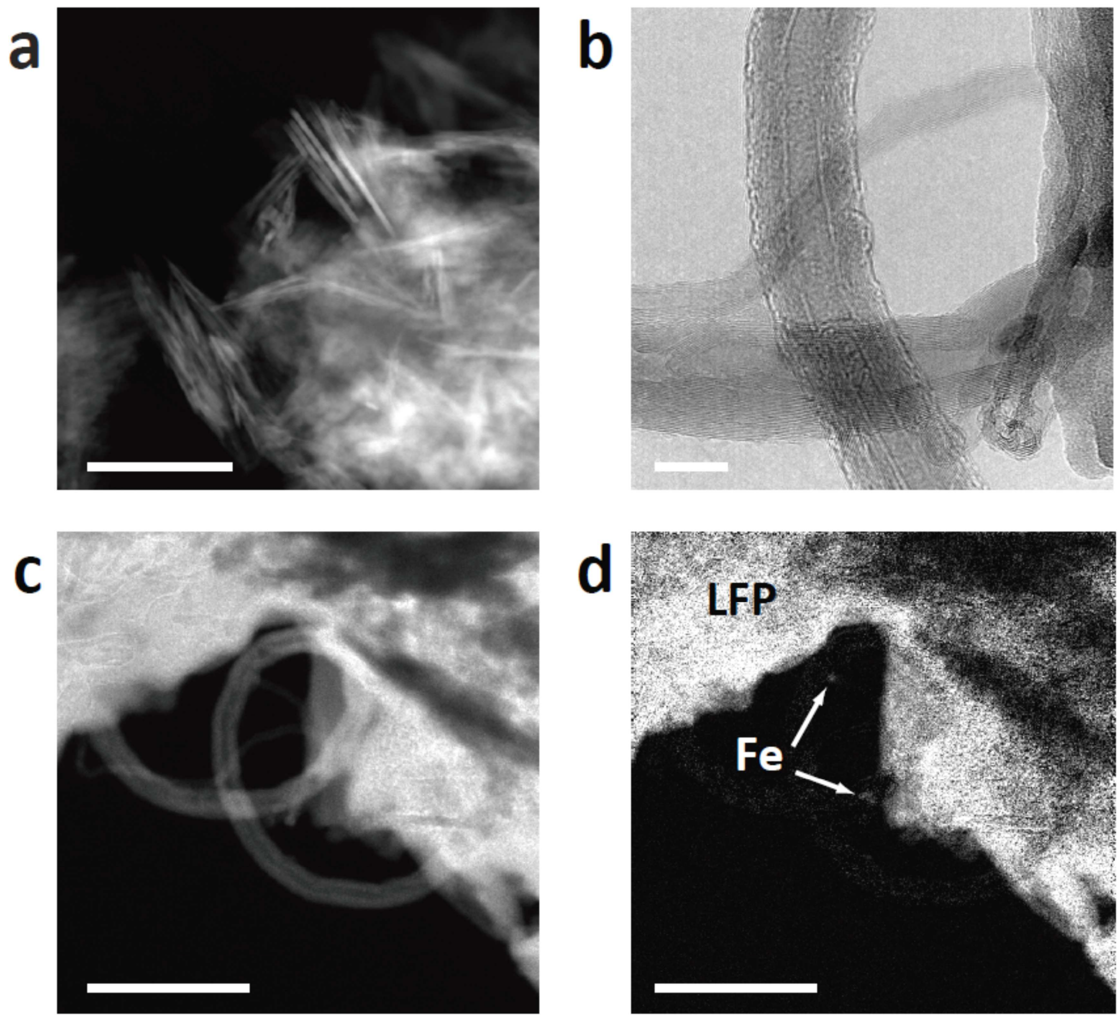

Supplementary Figure 16 STEM image of the film: (**a**) STEM images of the LFP nanoplatelets (scale bar 200nm). (**b**) HRTEM image showing the CNTs (scale bar 10 nm). (**c**) Filtered map from the plasmon region (before Li-K and Fe-M edges) evidencing the CNTs (scale bar 100 nm). (**d**) Map of the overlapping Li-K and Fe-M edges (scale bar 100 nm) from the same region in (**c**), revealing only the signals from the nanoplatelets and the small Fe particles in the CNTs (growth catalyst). No signs of Li compounds or Li intercalation in the CNTs were found.

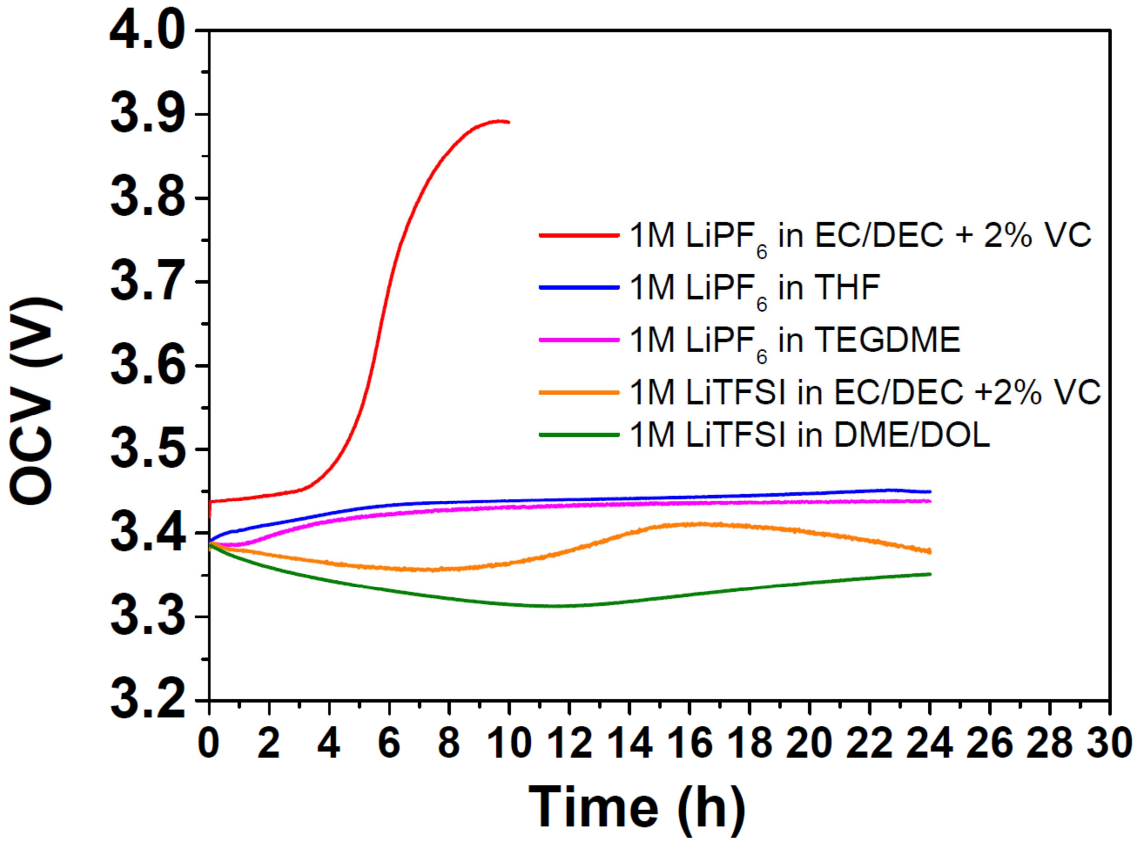

Supplementary Figure 17 Electrolyte dependence of the photo-oxidation: OCV curves for different electrolyte compositions

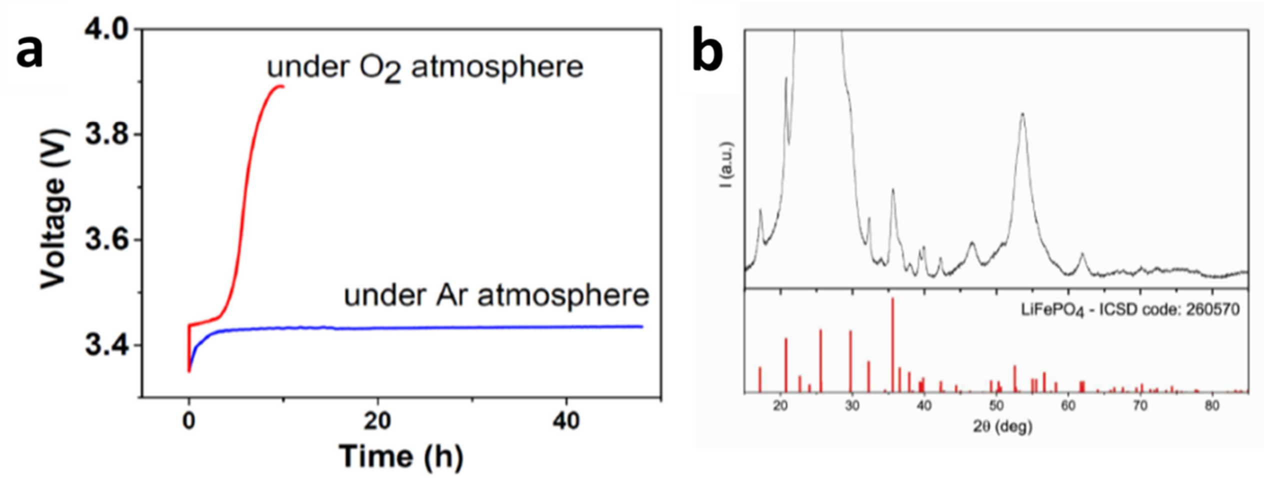

Supplementary Figure 18 Atmosphere dependence of the photo-oxidation: (**a**) OCV of the film under oxygen (red line) and argon (blue line), (**b**) XRD pattern of the film after OCV under Argon exposure

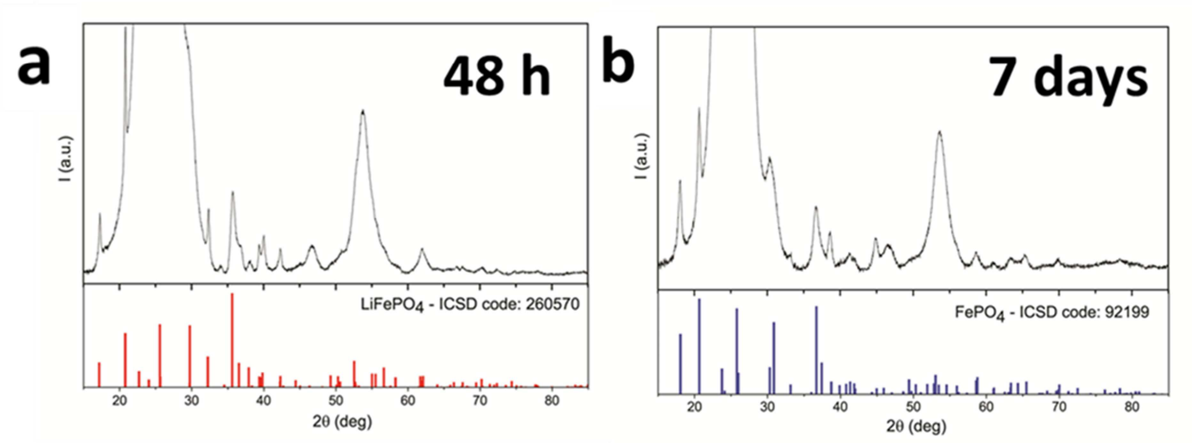

Supplementary Figure 19 XRD pattern of the films immersed in the electrolyte XRD patterns of $LiFePO_4$ after (**a**) 48 hours and (**b**) 7 days immersion in 1M $LiPF_6$ EC/DEC +VC electrolyte in the absence of lithium metal as counter-electrode.

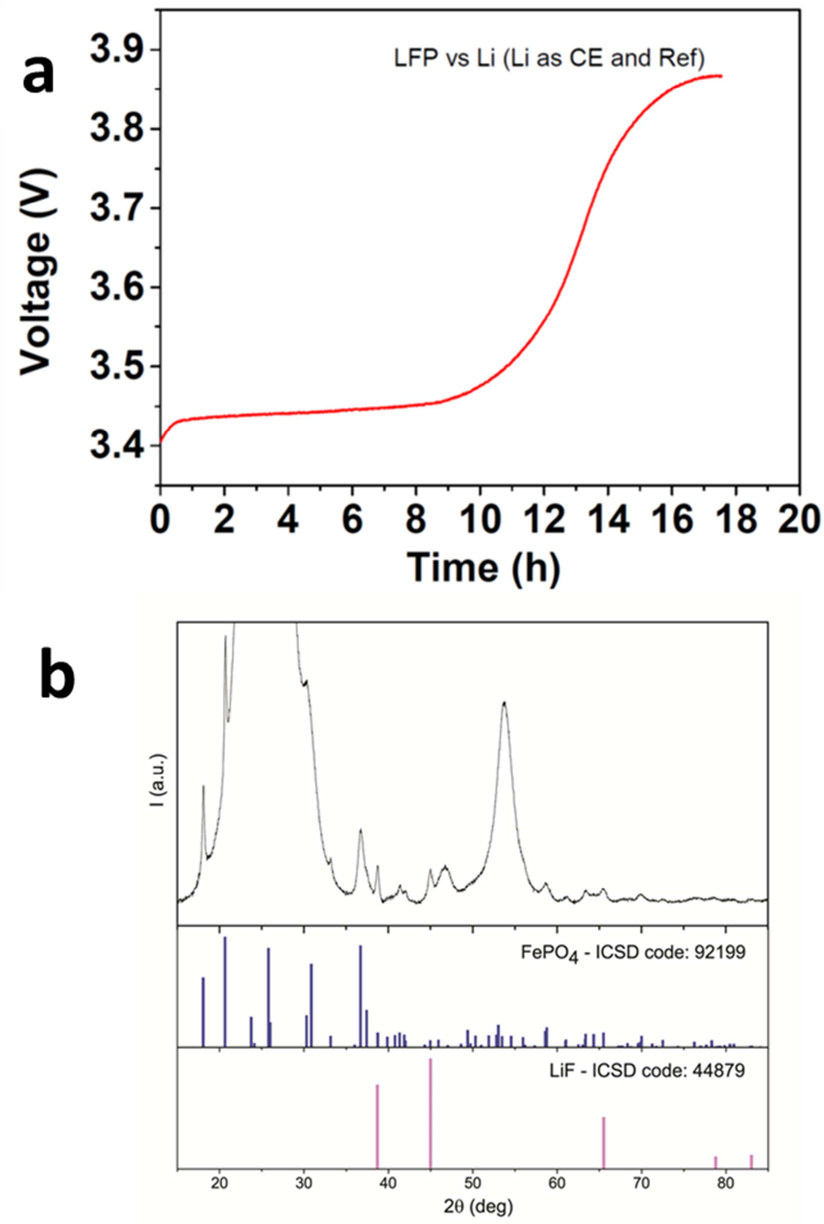

Supplementary Figure 20 Two electrode cells setup: (**a**) OCV curve of LFP photocathode vs. Lithium and (**b**) XRD pattern of the film after photo-oxidation.

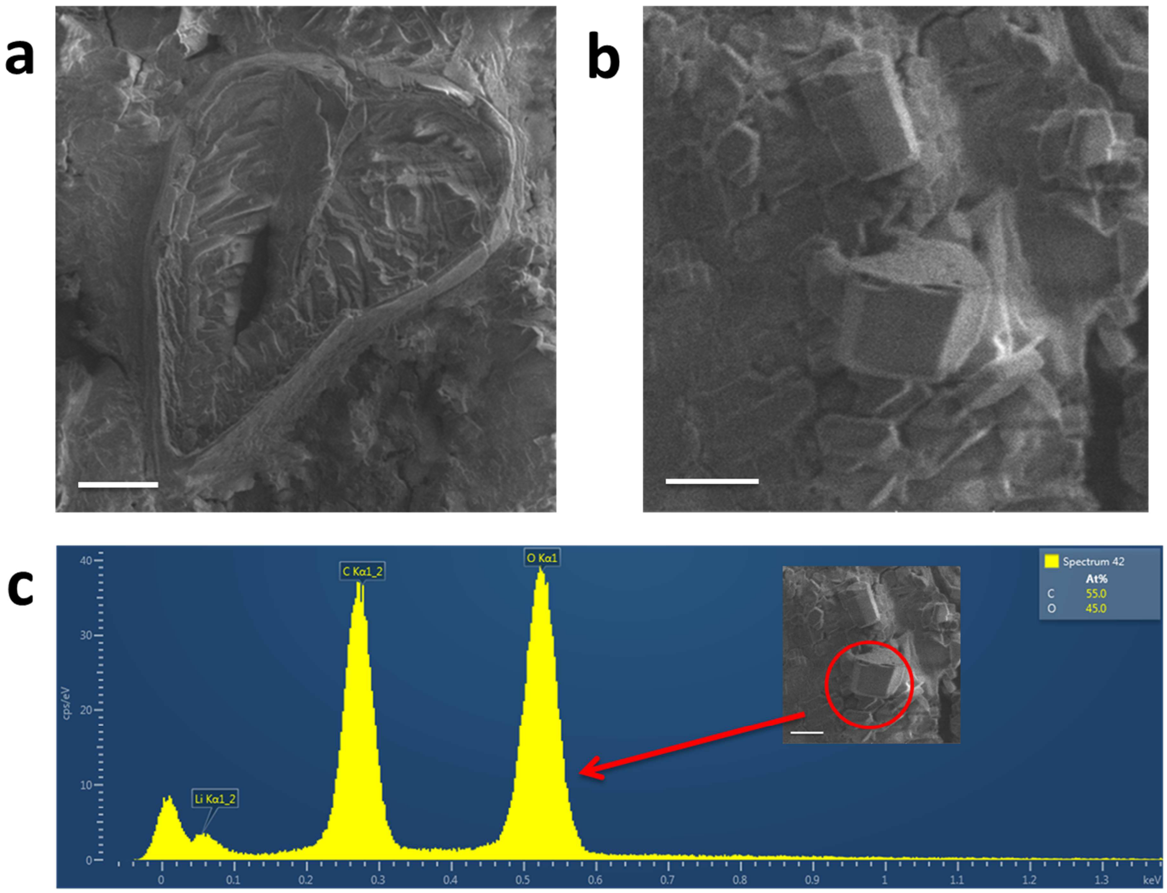

Supplementary Figure 21 SEM image and X-ray analysis of lithium surface: (**a**) (scale bar 10 µm) –(**b**) (scale bar 2 µm)Scanning electron microscope (SEM) images of crystals grown on the Li surface after photo-oxidation (charge state), (**c**) Local X-ray analysis[3] showing that the crystals are rich in Li, C and O.

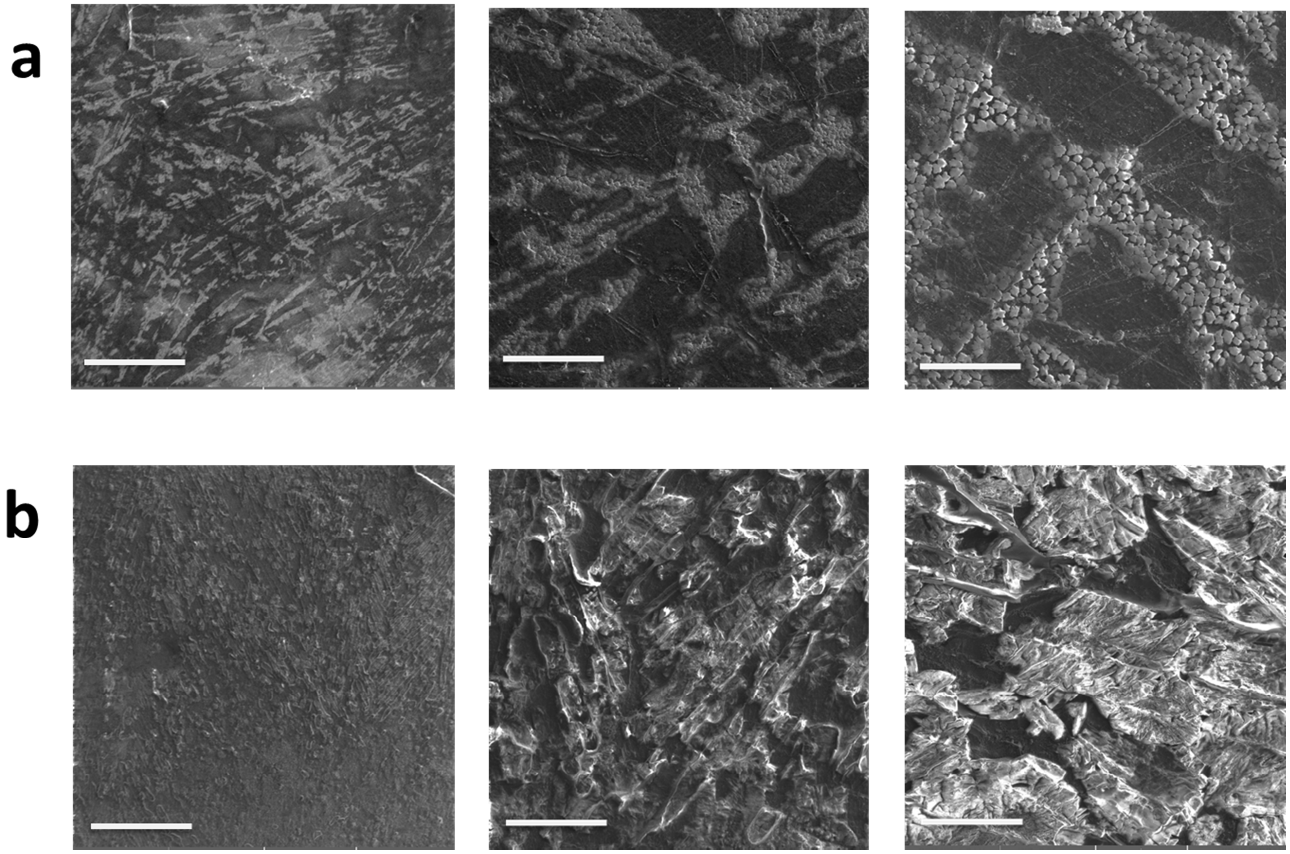

Supplementary Figure 22 Comparison of lithium surfaces with and without photocathode: Scanning electron microscopy (SEM) analysis of morphology of SEI formed on (**a**) lithium metal immersed in 1M LiPF6 in EC/DEC + 2% VC (scale bars respectively 500, 100, 50 µm) and (**b**) lithium metal immersed in 1M LiPF6 in EC/DEC + 2% VC in the presence of the film LFP/CNTs/N719 dye under light illumination (scale bars respectively 500, 100, 50 µm).

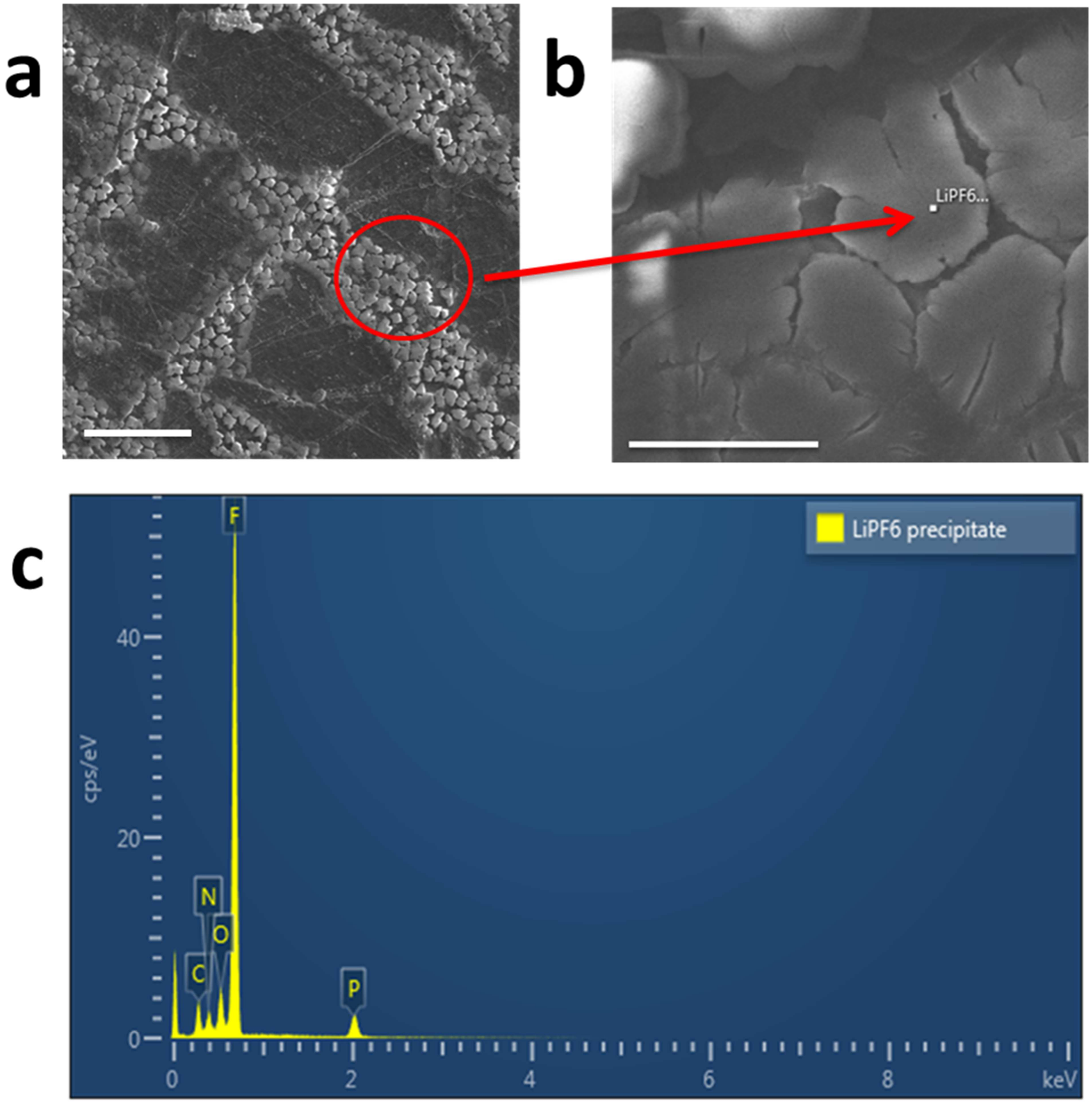

Supplementary Figure 23 SEM and X-ray analysis of Lithium surface: (**a**) (scale bar 50 µm) and (**b**) (scale bar 5 µm) Scanning electron microscopy (SEM) image of SEI of lithium metal immersed in 1M $LiPF_6$ in EC/DEC +2% VC and (**c**) compositional analysis showing the presence of carbonates and $LiPF_6$.

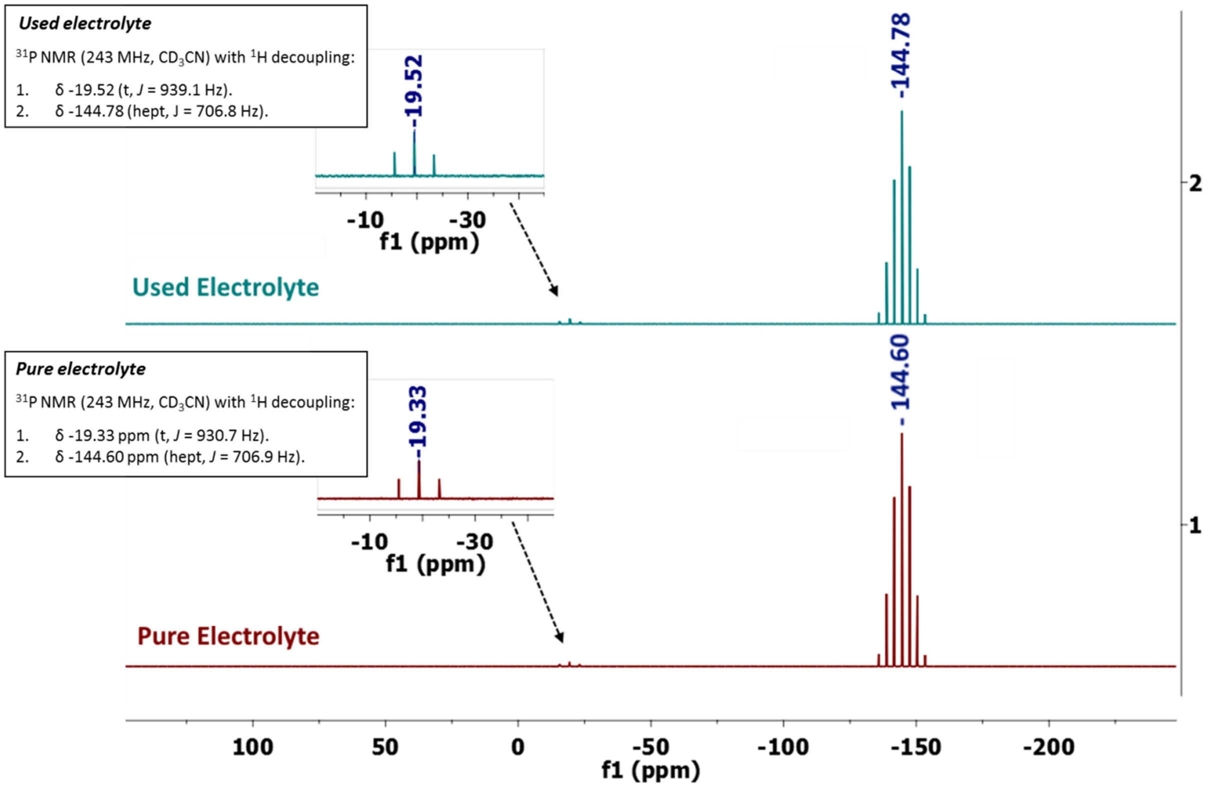

Supplementary Figure 24 Phosphorus NMR analysis of the electrolyte after cycling: ^{31}P NMR with ^{1}H decoupling spectra of the pure electrolyte (bottom) and used electrolyte after 1 cycle (top) in deuterated acetonitrile (CD_3CN). In each sample there is a septet at about -144ppm representing the ion PF_6^{-4}. There is also the presence of a triplet at about -19 ppm which is attributed to the normal $LiPF_6$ decomposition in electrolyte[5]. No other phosphorus species were detected.

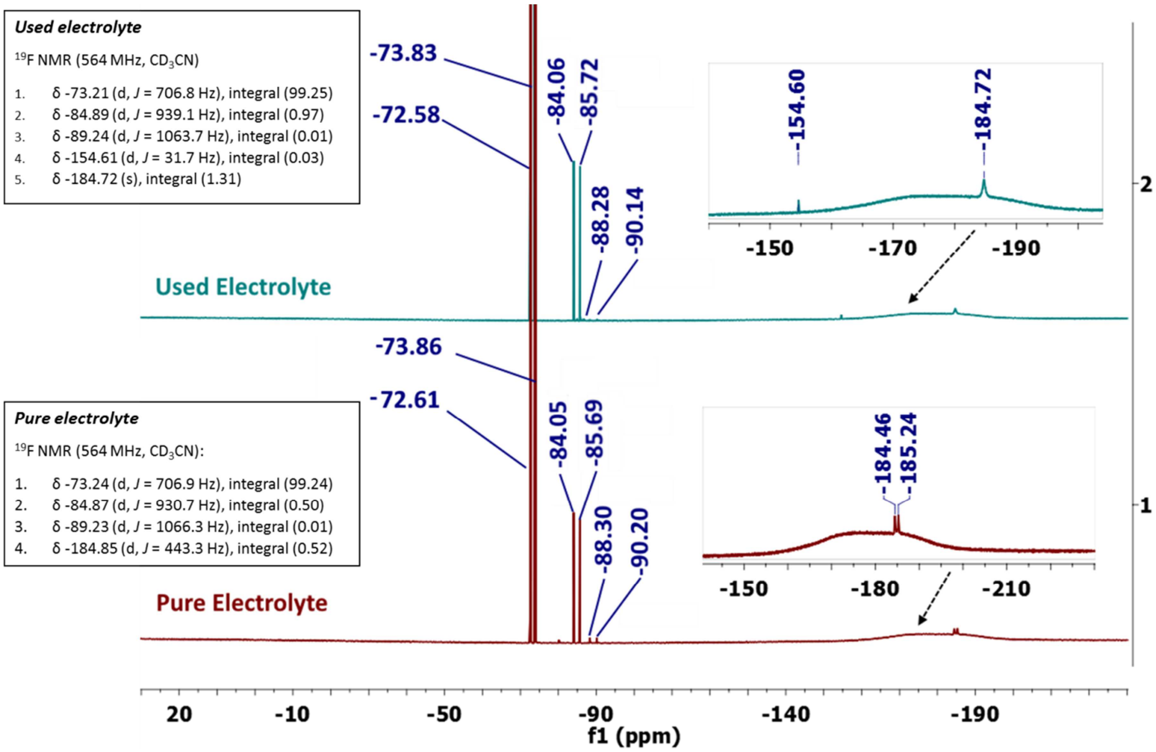

Supplementary Figure 25 Fluorine NMR analysis of the electrolyte after cycling: ^{19}F NMR spectra of the pure electrolyte (bottom), used electrolyte after 1 cycle (top) in deuterated acetonitrile (CD_3CN). In each sample there is a doublet at about -73ppm representing the ion PF_6^-. There is also the presence of a doublet at about -84 ppm and another very small at about -89 ppm which are all attributed to the normal $LiPF_6$ decomposition in electrolyte. There are also some traces of an unknown fluorinated compounds around -184 ppm. Traces of HF were detected after cycling as evident by the appearance of a peak at -154.60ppm[5].

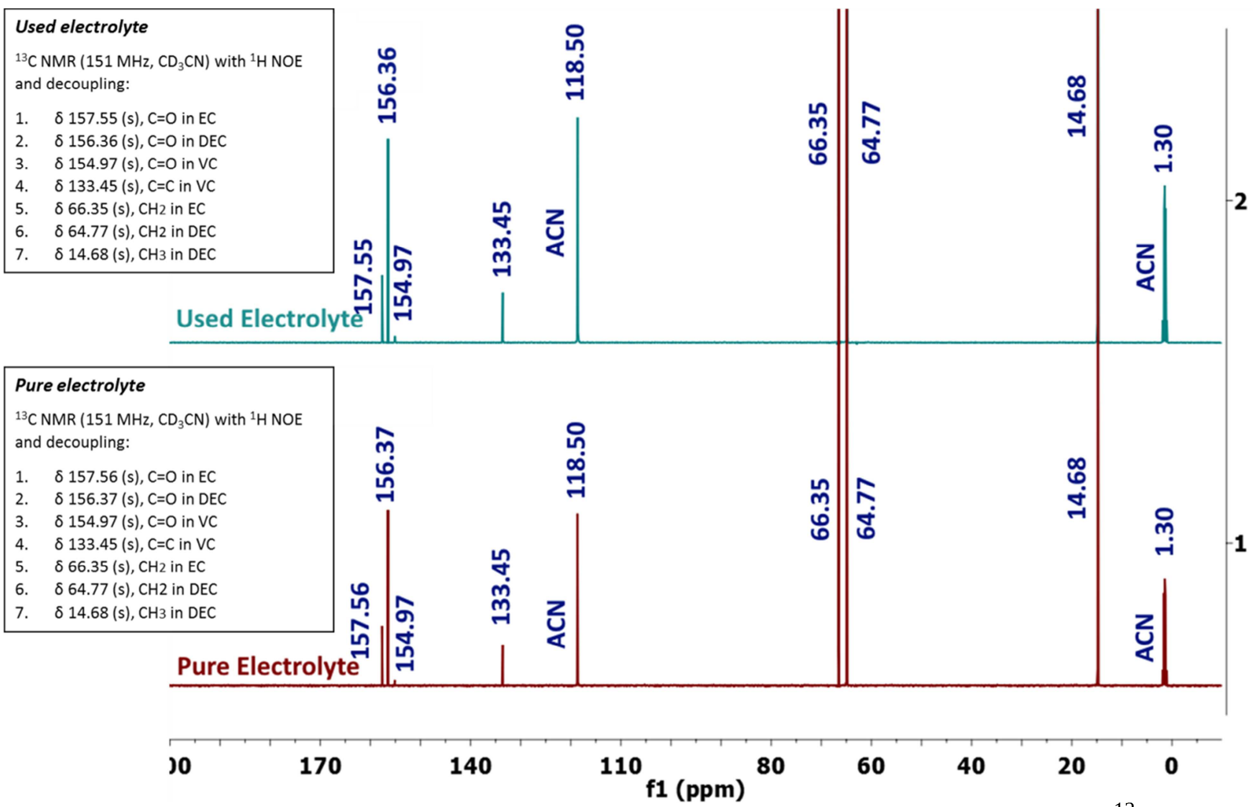

Supplementary Figure 26 Carbon NMR analysis of the electrolyte after cycling : ^{13}C NMR spectra of the pure electrolyte (bottom) and used electrolyte after 1 cycle (top) in deuterated acetonitrile (CD_3CN). NMR ^{13}C analysis does not reveal any evidences of the presence of new species formed in the electrolyte after one cycle.

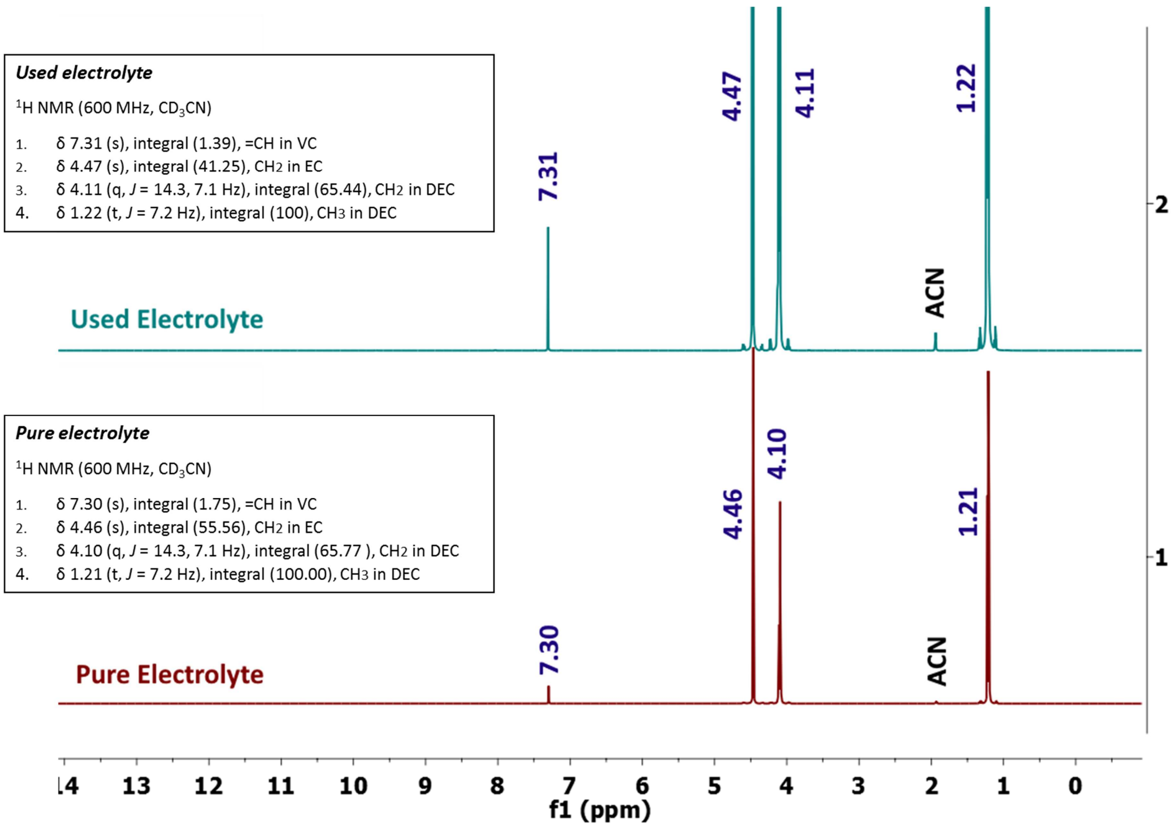

Supplementary Figure 27 Proton NMR analysis of the electrolyte after cycling: ^{1}H NMR spectra of the pure electrolyte (bottom) and used electrolyte after 1 cycle (top) in deuterated acetonitrile (CD_3CN). After cycling, few extremely small peaks difficult to distinguish from the background noise were present in an area smaller than 0.01% compared to the area of the peak of the electrolyte.

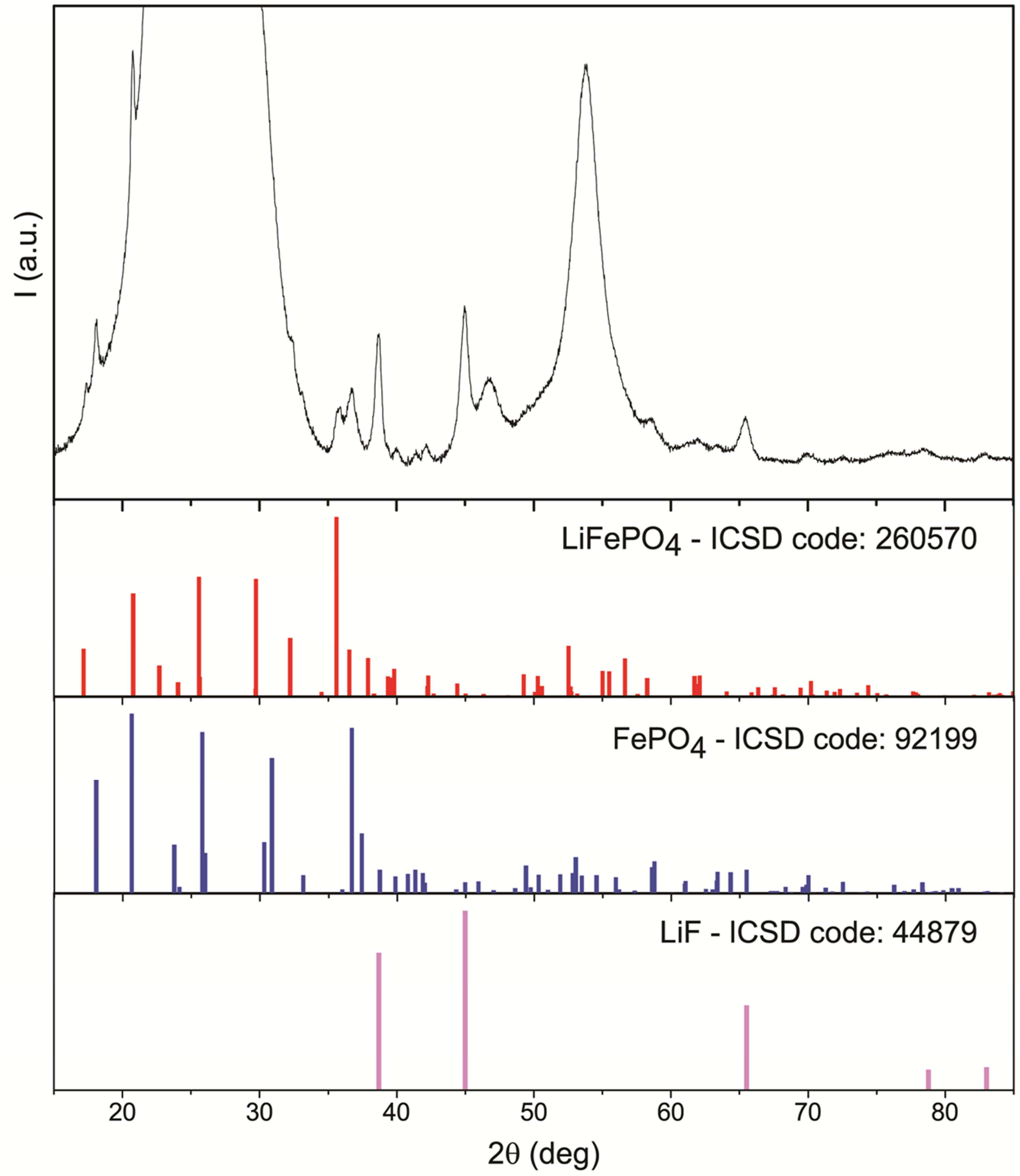

Supplementary Figure 28 XRD pattern of the film after discharge: XRD pattern of the film after 48 hours of discharge corresponding to twice the theoretical capacity. $LiFePO_4$ and $FePO_4$ are seen to co-exist

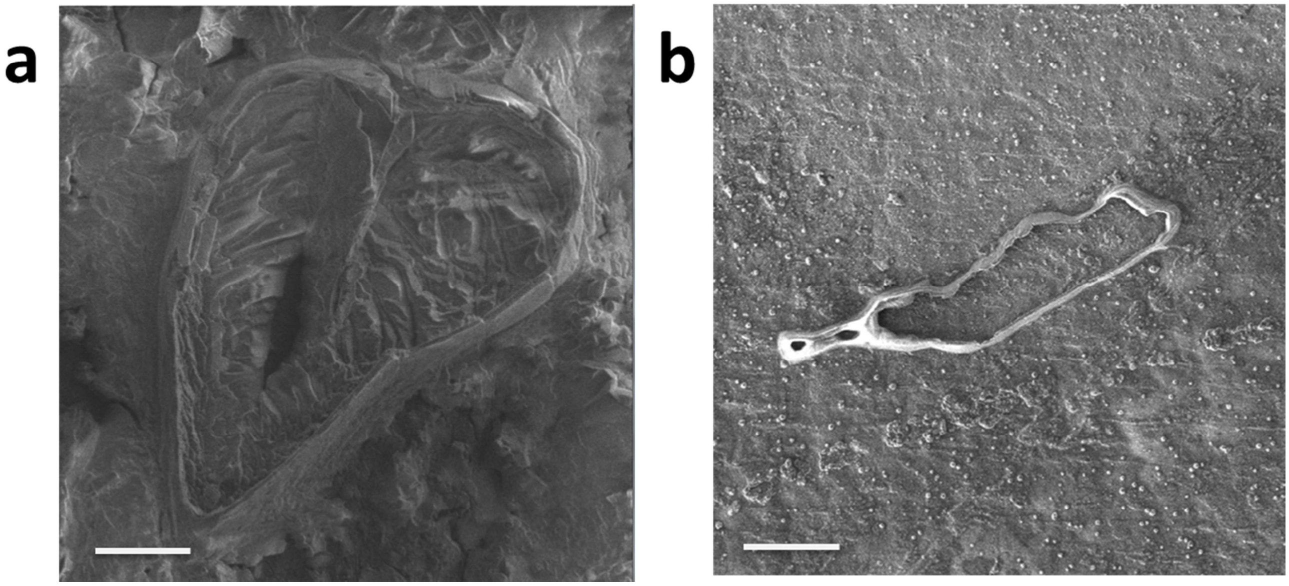

Supplementary Figure 29 SEM images of the lithium surface before and after discharge: (**a**) SEM micrographs showing Lithium after OCV illumination (scale bar 50 µm) and (**b**) after discharge (scale bar 20 µm). Only the outer ring of the initial crystal remains after discharging

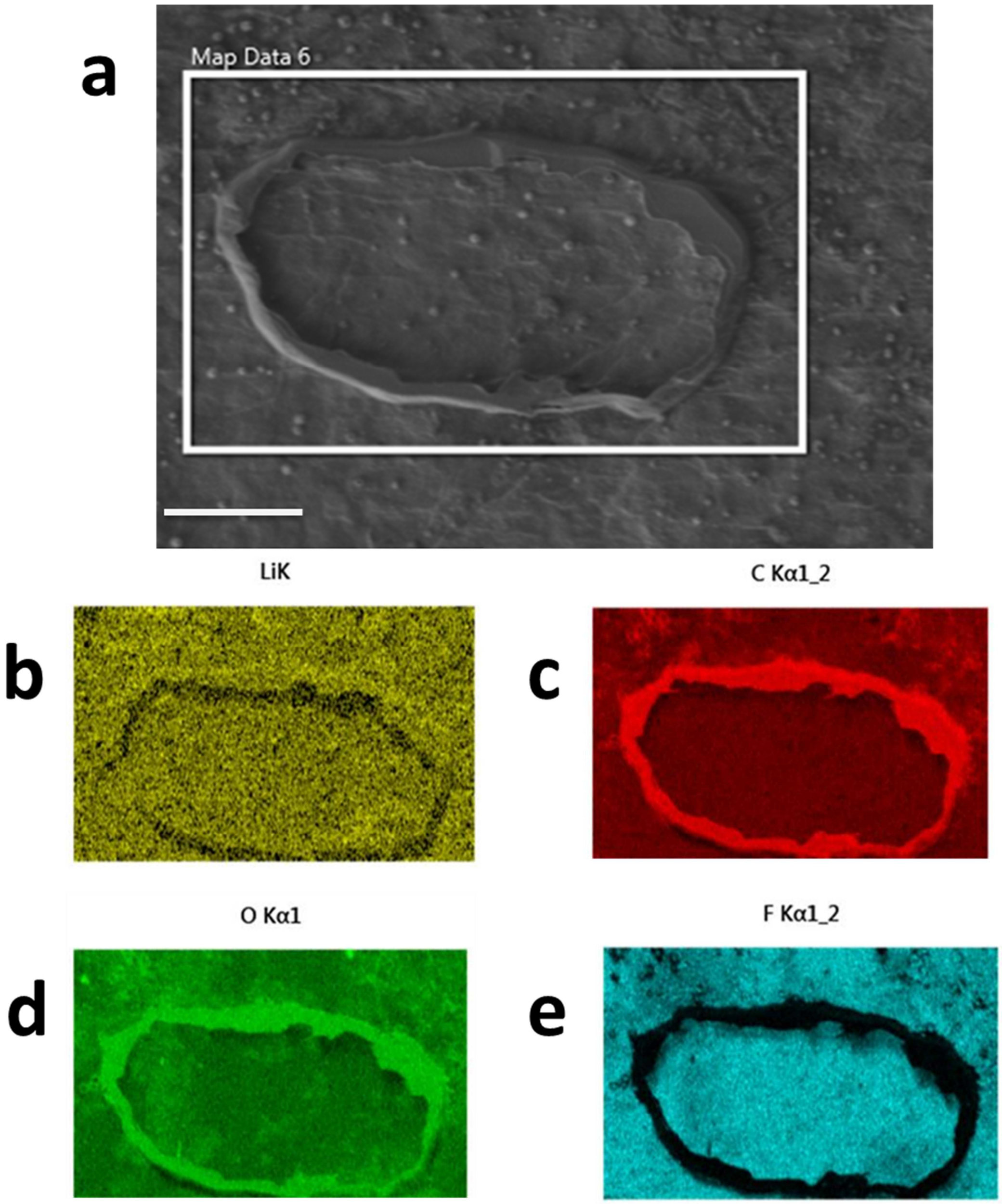

Supplementary Figure 30 TOF-SIMS analysis of lithium surface: TOF-SIMS analysis of lithium metal after discharge: (**a**) electron image (scale bar. 10 µm), (**b**) lithium, (**c**) carbon, (**d**) oxygen and (**e**) fluorine mappings

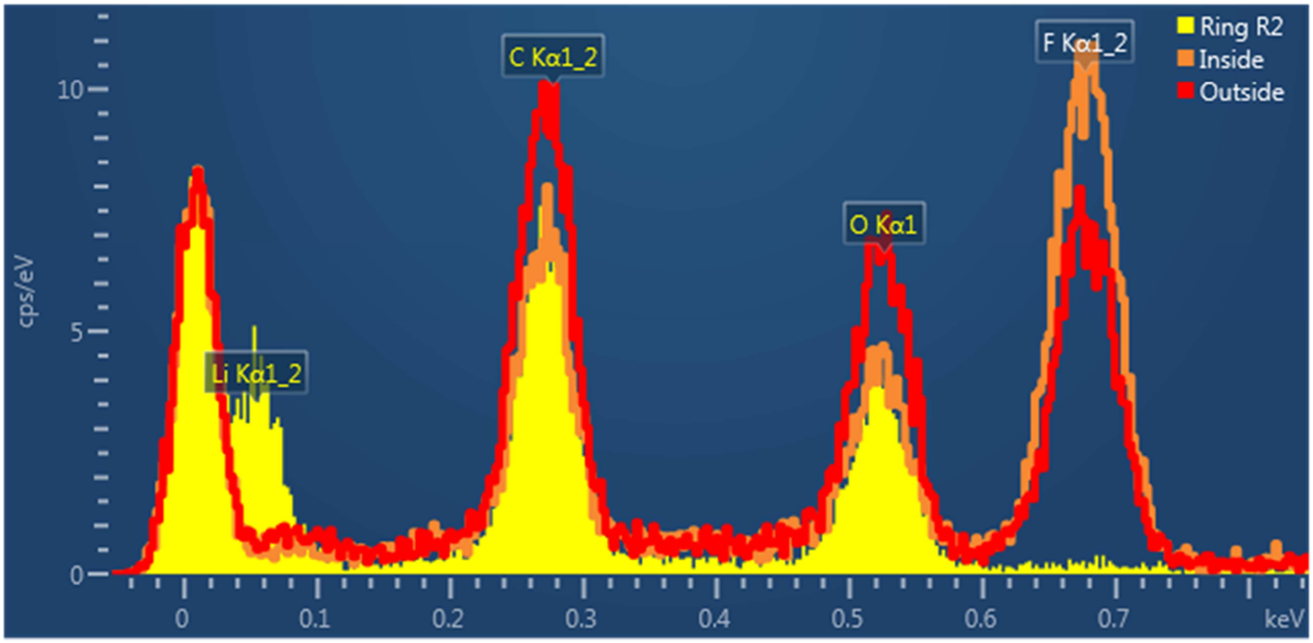

Supplementary Figure 31 Point elemental analysis Point analysis of the ring observed on lithium metal surface after discharge (**Supplementary Figure 29b**)

Supplementary Method 1

Energy conversion efficiency= Input (solar energy)/output (battery discharging)

Received solar energy by 11.8 h illumination (Input) = 2cm^2*100mW cm^-2*11.82h

Integration of the blue curve, area below= 63.3 h*V

Total discharge energy (output) = 63.3h*V*22.0μA

Therefore, Efficiency = (63.3h*V*22.0μA)/(2cm^2*100mWcm^{-2}*11.82h) = 0.06%

Supplementary Method 2

Faradaic charging Efficiency = Faradaic charging energy/solar illumination

mass of $LiFePO_4$ = 1.56 mg cm^{-2} × 2cm^2= 3.12 mg, according to Faraday's Law:

q=(m M^{-1})Fz, where z equals to 1 as the oxidation of Fe^{2+} to Fe^{3+} there is only one electron transferred, F equals to 96485 C mol^{-1} as Faraday constant.

Therefore, the oxidation of LFP/LP related charges q=1.89 C

Faradaic charging Efficiency =(1.89 C×3.6 V)/(2cm^2×100mW cm^{-2} × 11.82 h) = 0.08%

Supplementary References

1. Wilken, S.*et al.* Initial stages of thermal decomposition of LiPF6-based lithium ion battery electrolytes by detailed Raman and NMR spectroscopy. *RSC Adv.* **3,** 16359–16364 (2013).

2. Campion, C. L.*et al.* Thermal decomposition of LiPF6-based Electrolytes for lithium-ion batteries. *J. Electrochem. Soc.* **152,** A2327 (2005).

3. Hovington, P. et al. Can we Detect Li K X-ray in lithium compounds using energy dispersive spectroscopy? *Scanning* **38**, 571–578 (2016).

4. Lee, H. *et al.* The function of vinylene carbonate as a thermal additive to electrolyte in lithium batteries. *J. Appl. Electrochem.* **35,** 615–623 (2005).

5. Plakhotnyk, A. V. *et al.* Hydrolysis in the system LiPF6—propylene carbonate —dimethyl carbonate—H2O. *J. Fluor. Chem.* **126,** 27–31 (2005).
